# Enhancing Mechanical Resilience in Li-Ion Battery Cathodes with Nanoscale Elastic Framework Coatings

**DOI:** 10.1021/acsnano.4c14980

**Published:** 2025-01-03

**Authors:** Jong-Heon Lim, Jaehyun Kim, Jiwoong Oh, Jaesub Kwon, Kyoung Eun Lee, Youngsu Lee, Seongeun Park, Jun Lim, Dongwook Shin, Changshin Jo, Yong-Tae Kim, Janghyuk Moon, Mark C. Hersam, Kyu-Young Park

**Affiliations:** † Graduate Institute of Ferrous & Eco Materials Technology (GIFT), Pohang University of Science and Technology University, Pohang 37666, Republic of Korea; ‡ Department of Energy Systems Engineering, Chung-Ang University, Seoul 06974, Republic of Korea; § Department of Materials Science and Engineering (MSE), Pohang University of Science and Technology University, Pohang 37673, Republic of Korea; ∥ Pohang Accelerator Laboratory (PAL), Pohang University of Science and Technology, Pohang 37673, Republic of Korea; ⊥ Materials Development Group, Samsung SDI, Samsung Future Technology Campus, 130 Samsung-ro, Yeongtong-gu, Suwon, Gyeonggi 16678, Republic of Korea; # Department of Chemical Engineering, Pohang University of Science and Technology University, Pohang 37673, Republic of Korea; ∇ Department of Materials Science and Engineering, Northwestern University, Evanston, Illinois 60208, United States; ○ Department of Chemistry, Northwestern University, Evanston, Illinois 60208, United States; ◆ Department of Electrical and Computer Engineering, Northwestern University, Evanston, Illinois 60208, United States

**Keywords:** lithium-ion batteries, surface modification, carbon nanotube, elastic framework, mechanical resilience

## Abstract

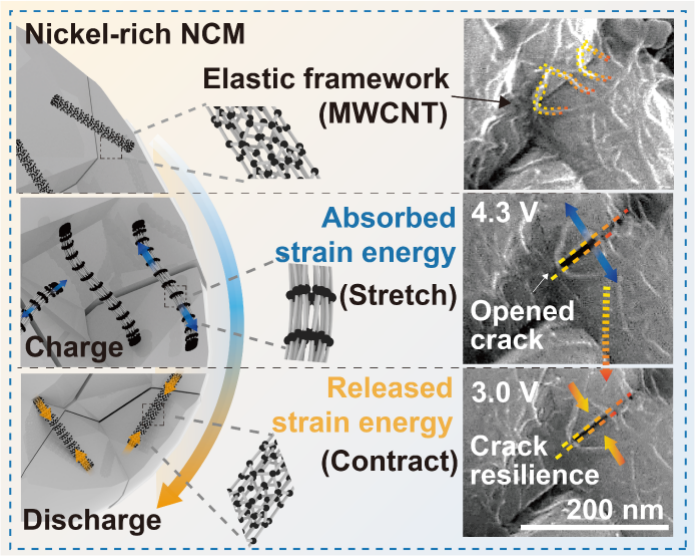

Lattice volume changes in Li-ion batteries active materials are unavoidable during electrochemical cycling, posing significant engineering challenges from the particle to the electrode level. In this study, we present an elastic framework coating designed to absorb and reversibly release strain energy associated with particle volume changes, thereby enhancing mechanical resilience at both the particle and electrode levels. This framework, composed of multiwalled carbon nanotubes (MWCNTs), is applied to nickel-rich LiNi_0.9_Co_0.05_Mn_0.05_O_2_ (NCM9055) cathodes at a low loading of 0.5 wt %, effectively mitigating critical issues such as particle cracking, volume changes, and electrode thickness variations during cycling. Leveraging these advantages, an energy-dense electrode is achieved with a high active material loading of 20 mg cm^–2^, without the need for additional carbon additives. Demonstrated in a pouch cell format, this electrode achieves an exceptional capacity retention of 77.7% after 1000 cycles. This approach provides a comprehensive solution for designing Li-ion batteries capable of withstanding lattice volume variations, offering valuable insights for next-generation batteries technologies.

## Introduction

Volume changes in active materials, driven by electrostatic interactions among atoms that vary with the degree of lithiation and delithiation, pose significant engineering challenges in lithium-ion batteries (LIBs) systems.^1,2^ These challenges critically affect cycle stability and safety, particularly for next-generation active materials such as silicon anodes and high-nickel oxide cathodes.^3,4^ During cycling, fluctuations in particle volume can damage the solid-electrolyte interphase (SEI) layer,^5^ isolate active particles,^6^ and cause particle cracking,^7^ all of which severely undermine the cycle retention of batteries. Moreover, in extreme cases, volume changes at the pack level can lead to serious safety hazards, such as thermal runaway triggered by the combined effects of current hot spots and lattice oxygen evolution.^8^

High-Ni multicomponent layered oxide cathodes (LiNi_1__–*x*–_*_y_*Co_*x*_Mn_*y*_O_2_, NCM), with a Ni ratio exceeding 80%, offer an energy density of up to ∼800 Wh kg^–1^, making them prime candidates for state-of-the-art electric vehicles (EVs) applications.^9^ However, the dynamic lattice changes during electrochemical cycling, particularly along the *c*-lattice, significantly impact cycle retention through chemical and mechanical degradation.^10^ While mild contraction is observed during the H1-M (3.5–3.8 V, ∼1% volume contraction) and M-H2 (3.8–4.1 V, ∼1% volume contraction) phase transitions, severe contraction occurs during the H2–H3 transition (>4.1 V, ∼4% contraction),^11^ resulting in a total lattice volume contraction of nearly 6% relative to the original volume.^12,13^ These lattice volume changes are primary initiators of mechanical cracking along grain boundaries, with crack propagation and accumulation intensifying through repeated cycling.^14^ Newly exposed surfaces then undergo chemical degradation, such as lattice oxygen evolution,^15^ fatigue degradation,^16^ rock-salt phase formation, and electrolyte decomposition.^17^

Prolonged cycling exacerbates these instabilities, as repeated volume changes compromise electrical contacts between active materials, conducting agents, and the current collector.^18^ Similarly, advanced high-energy-density electrodes, which demand higher active material loadings and reduced carbon and binder contents, face challenges due to electrode thickness changes, delamination, and active particle isolation.^13^ Collectively, these factors present significant barriers to the stability of nickel-rich cathodes, undermining their viability at both the particle and electrode levels.^19,20^

In an effort to mitigate particle-level challenges, various microstructure engineering strategies have been proposed.^21^ For instance, meticulous control over primary particle morphology has been achieved through the introduction of dopants,^22−25^ effectively impeding microcrack propagation by optimizing the distribution of fracture energy across particle surfaces. Furthermore, strategies to minimize surface reactions, such as tailored electrolyte designs, have been employed to reduce mechanical crack formation.^26,27^ Additionally, the synthesis of single crystals, which eliminates mechanically vulnerable grain boundaries, is a promising approach to improve cycling retention.^28,29^ While these microstructure engineering approaches have successfully delayed chemo-mechanical degradation at the particle level, significant challenges remain in developing high-energy-density electrodes with extended cycle life for nickel-rich cathodes.

Active particle volume fluctuations also pose critical challenges in electrode design, leading to spatially inhomogeneous reactions and compromised contact quality.^30−32^ These issues induce state-of-charge (SOC) heterogeneity in both the upper and lower regions of the electrode and result in localized current distributions during cycling. To date, these challenges have necessitated electrode designs incorporating relatively high carbon black ratios to increase active loading densities or adjustments to active material loading to preserve cycle stability (see Table S1).^33^ However, current electrode designs fall short of achieving desirable architectures, such as combining a commercially viable loading density of over 20 mg cm^–2^ with extremely low carbon content (<1 wt %), thereby limiting the intrinsic energy density of high-nickel cathodes.^34^ Moreover, cells with high active loading levels are sensitive to pressure fluctuations caused by volume variation in active materials. High pressures exceeding 0.2 MPa increase cell resistance by reducing ionic pathways within the electrode, elevating internal impedance.^35^ Conversely, low pressures can lead to misalignment of internal components due to vibrational shocks, further increasing cell resistance.^36^ These challenges undermine the consistency and cycle reliability of lithium-ion batteries.

Here, we introduce an alternative strategy to address mechanical resilience challenges from the particle to the electrode level by implementing an exterior elastic surface framework coating. This elastic framework, composed of multiwalled carbon nanotubes (MWCNTs) tightly adhered to the active material surface, is engineered to absorb mechanical energy during delithiation and release it reversibly during relithiation, thereby enhancing the mechanical resilience of particles during repeated electrochemical cycling. This approach effectively minimizes crack propagation and accumulation while reducing volume variation at the secondary particle level, which in turn mitigates changes in electrode thickness during cycling. Specifically, the 0.5 wt % elastic framework introduced on LiNi_0.9_Co_0.05_Mn_0.05_O_2_ (NCM9055) cathodes reduces particle-level volume contraction to 3.2%, compared to nearly 6% in bare NCM9055, and maintains electrode thickness variations to less than half of that observed in bare NCM. By leveraging the elastic and conductive properties of the framework, we have developed an advanced electrode design with a high loading density of over 20 mg cm^–2^ without additional carbon additives. This high-loading-density electrode design, which has traditionally posed significant challenges for nickel-rich layered oxides, achieves an impressive capacity retention of 77.7% after 1000 cycles in pouch cells. Furthermore, this approach is compatible with solution-based processing and conventional battery manufacturing, offering a practical pathway toward high-performance lithium-ion batteries.

## Results and Discussion

### Formation of Exterior Elastic Frameworks

High-nickel NCM9055, known for its significant lattice volume changes of approximately 6%, was selected to demonstrate the concept of an exterior elastic framework. The material used in this study consists of secondary particles with diameters of 3–5 μm, composed of ∼500 nm primary particles, as confirmed by field-emission scanning electron microscopy (FE-SEM, Figures S1 and 1A). Multiwalled carbon nanotubes (MWCNTs) were chosen for the exterior framework due to their exceptional elastic modulus (∼1800 GPa) and high electrical conductivity.^37^

**Figure 1:**
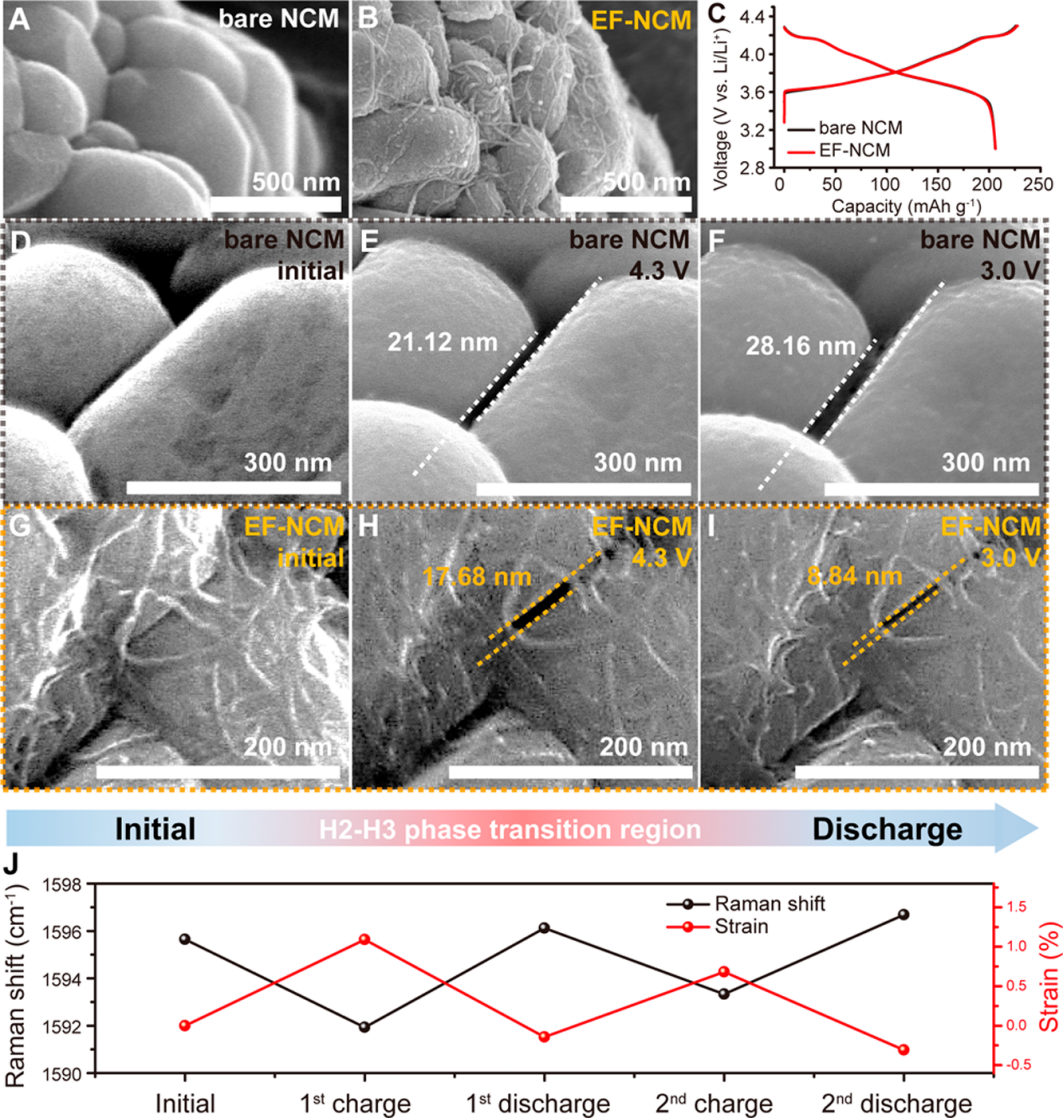
Observing the mechanical resilience of the elastic framework. FE-SEM analysis results of (a) bare NCM and (b) EF-NCM. (c) GCD curve of 1st charge/discharge process. Surface observation result of (d–f) bare NCM and (g–i) EF-NCM during charge/discharge process. (j) Statistical Raman analysis of CNT formed on EF-NCM during repeatable cycle process.

To form a tightly bonded MWCNT framework on the particle surface, a modified Pickering emulsion coating process involving ethyl-cellulose (EC) pyrolysis was employed (Figure 1B), with details provided in the Experimental Section. The target proportion of the elastic framework (0.5 wt % coated NCM) was optimized based on electrochemical performance, as shown in Figures S2 and S3. Thermogravimetric analysis (TGA) and Raman spectroscopy confirmed the presence of the elastic framework, as shown in Figures S4 and S5 (hereafter referred to as elastic framework NCM, EF-NCM).

Subsequent field-emission transmission electron microscopy (FE-TEM) revealed that the MWCNTs formed an exterior framework with a thickness of ∼10 nm (Figure S6). It was confirmed that low-temperature annealing of EC at 240 °C enhanced CNT adhesion through the formation of amorphous carbon.^38−40^ A detachment test was conducted on EF-NCM using probe sonication at energy levels of 800, 4000, and 8000 J per 0.05 g of NCM particles to confirm the stable adhesion of MWCNTs to the active surface (Figure S7). (Additional attachment test results, including samples sonicated in electrolyte and after cycling, are provided in Figure S8.) Furthermore, X-ray diffraction validated the intact crystal structure of NCM9055 after the application of the elastic framework (Figure S9). Collectively, these characterizations confirm that the elastic framework is securely bonded to the particle surface without causing structural damage to the NCM9055.

### Crack Resilience Effect of Elastic Framework

A comparative analysis of crack evolution behavior between EF-NCM and bare NCM was conducted by tracking grain boundaries using FE-SEM. For this analysis, electrodes were designed with 5 wt % carbon, a loading density of 3 mg cm^–2^, and subjected to a low current density of 0.1 C to minimize reaction inhomogeneity. Initially, galvanostatic charge–discharge profiles were measured for both active materials, revealing consistent charge and discharge capacities of 227 and 206 mAh g^–1^, respectively (Figure 1C), indicating comparable electrochemical activity. At the initial state, the primary particles of both materials exhibited seamless interconnections (Figure 1D,G). However, during delithiation, bare NCM began to show the crack initiation (Figure 1E), which persisted even after the first discharge cycle (Figures 1F and S10).^41^ EF-NCM showed similar crack initiation after charging (Figure 1H), but a clear distinction emerged during the subsequent discharge process (Figure 1I). Specifically, the distances between primary particles were reversibly diminished after the first discharge, demonstrating more recoverable behavior compared to bare NCM (Figure S11). This crack resilience behavior was also observed during the second charge–discharge cycle (Figure S12).

**Figure 2:**
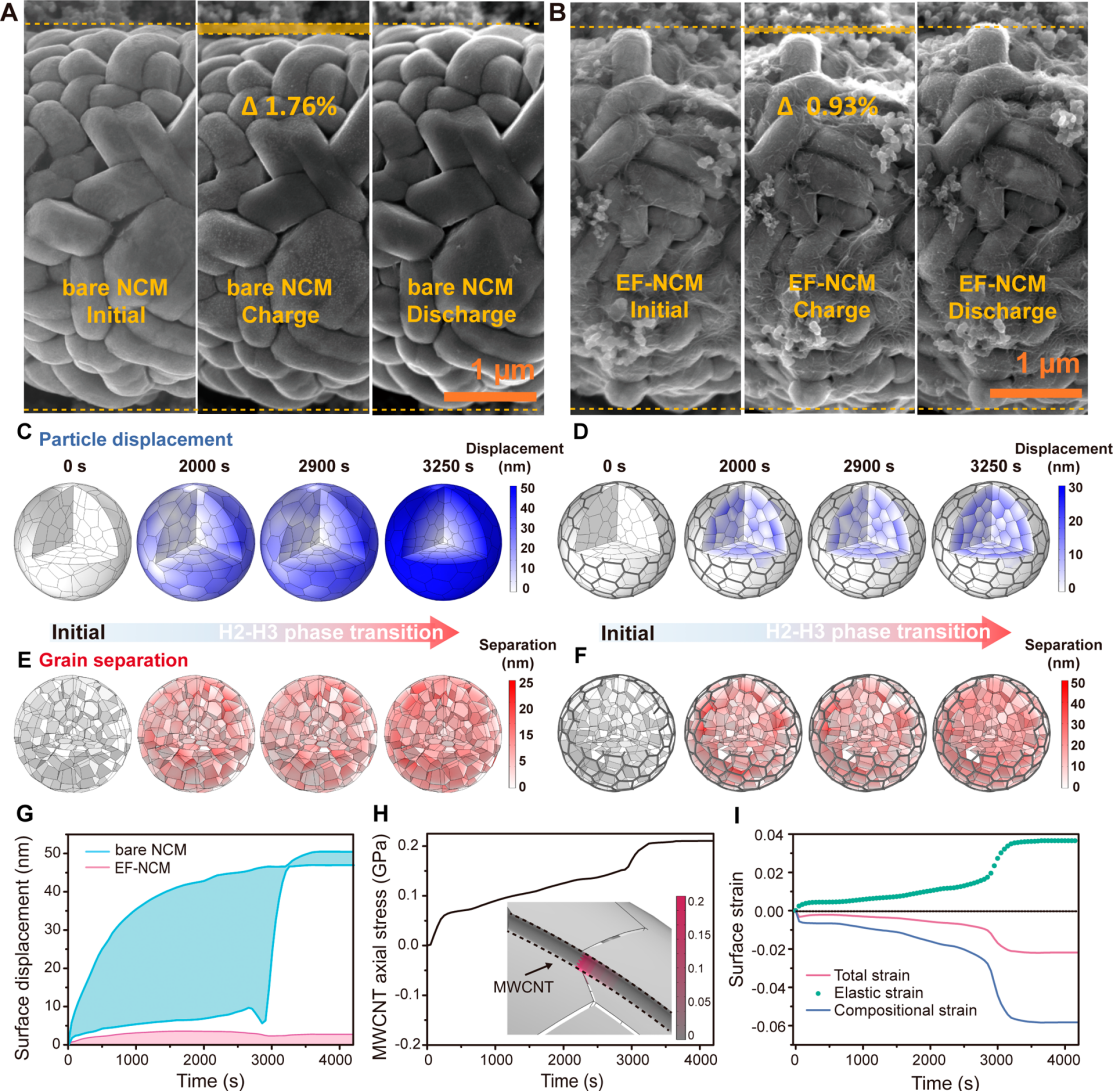
Particle volume tracking and computational simulations. Particle volume observation result of (a) bare NCM and (b) EF-NCM. Particle contraction and grain separation result from finite element analysis of (c, e) bare NCM and (d, f) EF-NCM. (g) Displacements at the surface of both bare NCM and EF-NCM. (h) Average axial stress generated within the MWCNT framework during charge process. (i) Strain analysis result of EF-NCM during charge process.

Raman spectroscopy was employed to further investigate the elastic behavior of the MWCNT framework by analyzing changes in doubly degenerate vibrational modes resulting from stress–strain interactions. To ensure statistically reliable Raman shift values, results from three separate analyses were averaged (detailed information is provided in Figure S13). Additionally, the Raman G-band exhibited the inherent characteristics of MWCNTs, without interference from polyvinylidene fluoride (PVDF) or residual EC (Figure S14). After charging to 4.3 V, the G-band of MWCNT shifted to lower wavenumbers, from 1595.6 to 1593.3 cm^–1^ (Figure 1J), and then reversibly returned to 1595.5 cm^–1^ upon discharging to 3.0 V. This observation suggests that tensile stress applied to the MWCNTs induces strain, leading to a separation of the doubly degenerate E2g and E1u vibrational modes, resulting in a subtle G-band shift.^42,43^ Strain calculations revealed a variation of less than +1%, with the detailed methodology provided in the supplementary text (Figure S13). The differences in Raman shift were further discussed in conjunction with computational calculation results (Figure 2C–F).

To further validate the ability of elastic frameworks to absorb mechanical energy, we conducted a nanoindentation test. The results revealed that EF-NCM exhibited a critical fracture load of approximately 2 ± 0.5 mN, which is twice as high as that of bare NCM9055 (∼1 ± 0.5 mN). This indicates that EF-NCM possesses significantly greater initial stiffness. (A detailed discussion of this result is provided in Figure S15.) The consistent behavior of the Raman shift over multiple charge–discharge cycles demonstrates the capacity of the MWCNT framework to absorb mechanical energy during charging, mitigating crack accumulation. Additionally, this absorbed energy is released during discharge, enhancing resilience against crack propagation.

### Suppression of Particle Volume Change

To gain additional insight into particle volume changes, a carbon-rich electrode was prepared with 20 wt % carbon to ensure electron percolation pathways. Additionally, to avoid initial mechanical damage, the calendaring process was omitted.^44^ Despite these unusual processing conditions, the galvanostatic charge–discharge graph of this carbon-rich, calender-free electrode exhibited a reasonable first discharge capacity of approximately 200 mAh g^–1^ at a current density of 0.1 C in constant current/constant voltage (CC/CV) mode (detailed electrode fabrication conditions are provided in Figure S16). *In-situ* X-ray diffraction (XRD) results indicated identical lattice volume variations in both bare NCM and EF-NCM electrodes, with volume contraction ratios of approximately 6% and a deviation of 0.01% after charging to 4.3 V (Figure S17). However, dramatic differences in secondary particle volume variations were observed. Bare NCM exhibited a particle diameter reduction of approximately 1.76% following charging to 4.3 V (Figure 2A). Statistical analysis revealed a contraction of nearly 6% in particle volume after charging, consistent with previous computational studies.^45^ In contrast, EF-NCM demonstrated a particle diameter shrinkage of only 0.93% in the charged state (Figure 2B), resulting in a particle volume contraction of just 3.2% (detailed statistical analysis results are described in Figure S18). Furthermore, after discharge, the particle volume of EF-NCM was reversibly restored to its original state. Additionally, while particle volume contraction in nickel-rich NCM typically leads to contact loss within the electrode,^46^ EF-NCM exhibited well-maintained electrical contacts (Figure S19). These results indicate that the elastic framework significantly reduces volume contraction and mitigates contact loss, thereby enhancing the electrochemical interface stability within the electrode structure.

Although Ni-rich materials exhibit volume contraction during charging, the elastic framework was unexpectedly found to demonstrate resilience under tensile stress, as indicated in the Raman results (Figure 1J). To better understand how this mechanical force influences the suppression of volume contraction in secondary particles, finite element analysis (FEA) was employed to elucidate the role of the elastic framework during electrochemical cycling. EF-NCM was modeled as a secondary active particle with an MWCNT framework firmly bonded to its surface, coupling beam and solid elements (Figure S20). The FEA compared the volume contraction behavior of EF-NCM with bare NCM under a moderate current density of 1 C, incorporating mechanical and lithium diffusion kinetics analyses of active particles. Lattice expansion coefficients, lithium diffusivity, and changes in lattice structure correlated with XRD and galvanostatic intermittent titration technique (GITT) results are shown in Figure S21, with additional parameters outlined in Table S3.

Displacements and grain boundary separations in the primary particles of both bare NCM and EF-NCM during charging were analyzed to evaluate secondary particle contraction and microcrack formation, respectively (Figure 2C–F). The simulated charging process corresponded to a capacity of 200 mAh g^–1^ over 3600 s, followed by a 600 s rest period to stabilize volume. Bare NCM exhibited a particle displacement of approximately 50 nm in its radius, which increased significantly during the H2–H3 phase transition at 3250 s (Figure 2C). This simulated surface displacement matched the 6% volume contraction observed from statistical volume tracking and XRD analysis (Figures S17 and S18). Conversely, EF-NCM showed a reduced particle displacement of only 20–30 nm, primarily in primary particles located in the inner part of the secondary particle (Figure 2D). Grain boundary separation results (Figure 2E) indicated that bare NCM, without contraction constraints, exhibited a small grain separation of 25 nm. In contrast, EF-NCM displayed a grain separation of nearly 50 nm at the charged state. This simulation suggests that the elastic surface framework effectively minimized secondary particle contraction through a volume-pinning effect by absorbing mechanical stress. However, as a part of a rebound effect, EF-NCM demonstrated a grain separation of 50 nm at 3250 s, which was also clearly observed in cross-sectional SEM images (Figures S22 and S23). Despite this rebound effect, overall particle displacement in EF-NCM was significantly reduced due to the elastic framework, ensuring sustained long-term cycling with minimal volume change.

The surface displacements of both bare NCM and EF-NCM were simulated to assess particle surface behavior during volume contraction (Figure 2G). The color-filled areas indicate the distribution of surface displacement for bare NCM (cyan) and EF-NCM (pink), spanning maximum to minimum displacement values of primary particles. Bare NCM exhibited an average contraction of 48 nm after 4200 s, attributed to lithium deintercalation from the NCM9055 particle. In contrast, EF-NCM displayed a significantly reduced surface contraction, with shrinkage at the EF-NCM surface being less than 10% of that observed in bare NCM9055. The MWCNT framework exhibited a positive axial strain (indicating tensile stress) of approximately 0.2 GPa (Figure S24), which rapidly increased during the H2–H3 phase transition (Figure 2H). This strain generation indicated that the elastic framework absorbed mechanical energy during particle contraction, preventing significant particle displacement.

Further strain component analyses of bare NCM and EF-NCM were conducted to understand the volume-pinning effect facilitated by the elastic framework. Bare NCM exhibited a strain component solely due to compositional strain (resulting from volume contraction), showing a 6% total contraction with no elastic strain (Figure S25). In contrast, EF-NCM experienced a 6% contraction due to delithiation but also exhibited a 4% expansion at the secondary particle level due to elastic strain (Figure 2I). The elastic strain provided by the framework effectively mitigated volume contraction, resulting in a total volume contraction of approximately 2% during 1 C charging. This strain analysis corroborates the statistical particle volume tracking results (Figure S18), further validating the effectiveness of the elastic framework in enhancing the volume retention of NCM9055.

### Enhanced Cycle Stability from Mechanical Resilience

**Figure 3:**
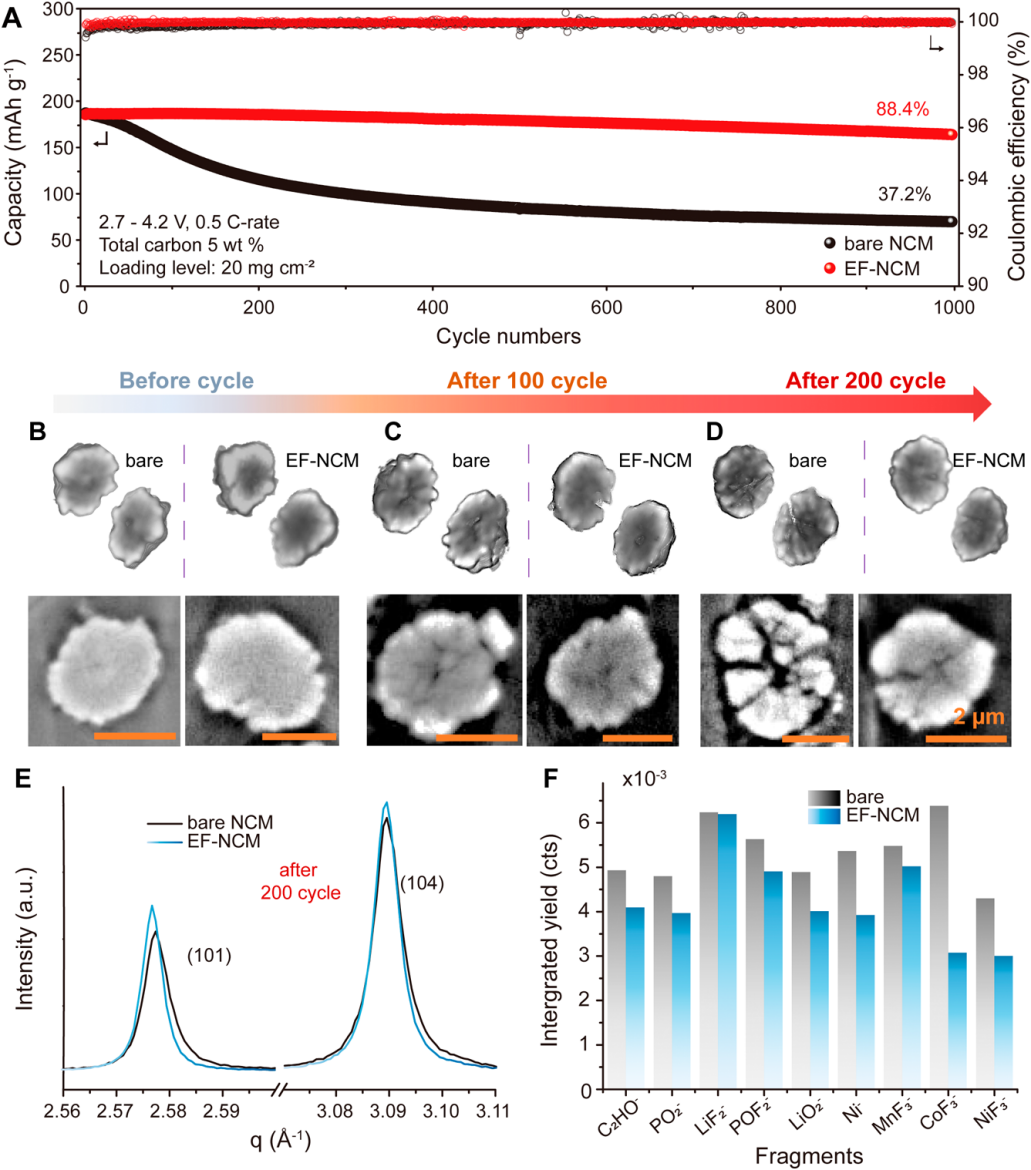
Cycling performance and postmortem analysis. (a) Long-term cycling of elastic framework modified NCM9055 with 5 wt % carbon additives. TXM analysis result of bare NCM and EF-NCM (b) before cycling, (c) after 100 cycles, and (d) after 200 cycles. (e) HRPD result of bare NCM and EF-NCM after 200 cycles. (f) ToF-SIMS results for bare NCM and EF-NCM.

The impact of mechanical resilience provided by the elastic framework on long-term batteries operation was evaluated. Specifically, a pouch cell was assembled with a commercially relevant loading density (20 mg cm^–2^) using an electrode containing 5 wt % conductive carbon. This carbon-rich condition ensured sufficient electron percolation to prevent reaction inhomogeneity,^52,53^ allowing clear differentiation of the effects of the elastic framework from the conductivity of MWCNTs. EF-NCM exhibited an outstanding cycle retention of ∼88.4% after 1000 cycles, demonstrating superior capacity retention compared to bare NCM (∼37.2%, Figure 3a). Notably, bare NCM electrodes exhibited a rapid decline in activity within the first 200 cycles, likely attributed to mechanical degradation caused by particle fracture (further discussed later). This superior cycle life was exclusively observed in EF-NCM electrodes, unlike control electrodes such as CNT-mixed and high-carbon-ratio (10% carbon black) designs (detailed in Figure S26). These findings highlight the critical role of crack resilience and volume pinning for achieving long cycle life in LIBs.

Nickel-rich cathodes with a secondary particle morphology are prone to mechanical degradation, leading to internal crack accumulation and eventual particle fracture during extended cycling.^54,55^ However, EF-NCM, decorated with a surface elastic framework, exhibited distinctive behavior characterized by internal space generation and volume pinning. Transition X-ray microscopy (TXM), a nondestructive imaging technique, was employed to visualize crack formation in electrodes after 200 cycles.^56^ Before cycling, cross-sectional CT imaging revealed a pristine surface devoid of cracks (Figure 3B). After 100 cycles, both bare NCM and EF-NCM showed minor crack formation (Figure 3C). However, after 200 cycles, bare NCM9055 exhibited significant crack accumulation and mechanical fractures resulting from repetitive charge–discharge processes (Movie S1), while EF-NCM maintained well-connected primary particle structures within the secondary particles (Figure 3D and Movie S2).

High-resolution powder diffraction (HRPD) analysis further revealed contrasting degradation conditions between bare NCM and EF-NCM after 200 cycles (Figure 3E). Specifically, for bare NCM, the (003) peak, indicative of the *c*-lattice slab distance, exhibited a lower peak position and increased broadening compared to EF-NCM (additional HRPD results are provided in Figure S27). This behavior suggests that bare NCM experienced an inhomogeneous charge state, likely due to particle fracture.^57^ Additionally, during long-term cycling, a shift in the (101) peak position toward higher values was observed in bare NCM, indicating contraction of the *a*-lattice and state-of-charge (SOC) heterogeneity within the secondary particles.^58^ Improved contact properties of EF-NCM, in contrast, enabled more reversible oxidation state changes, as confirmed by X-ray absorption near edge structure (XANES, Figure S28).

Time-of-flight secondary ion mass spectrometry (TOF-SIMS) analysis of the 2 nm surface region (Figure 3F) revealed a higher concentration of cobalt, nickel, and manganese ions in bare NCM compared to EF-NCM, despite comparable levels of electrolyte decomposition products such as LiF_2_^–^ between the two samples.^59^ XPS analysis further revealed clear lattice oxygen signals in EF-NCM after 100 cycles, confirming the thin SEI layer thickness (Figure S29). These results suggest that the SEI composition resulting from electrolyte decomposition in EF-NCM closely mirrored that of bare NCM. However, reduced transition metal dissolution was observed in EF-NCM after 200 cycles, underscoring the influence of mechanical resilience, as evidenced by TXM (Figure 3D) and XRD (Figure 3E) analyses. Collectively, these observations elucidate the surface-pinning effect of the elastic framework, which effectively suppresses irreversible crack formation and transition metal dissolution, thereby enhancing cycle stability.

### Achieving Advanced Electrode Design for Superior Energy Density

The mechanical resilience of the elastic framework enables advanced electrode designs characterized by low carbon ratios and commercial-level electrode densities. In this study, the MWCNT framework was exclusively utilized as a carbon additive to fabricate an advanced electrode architecture with a high active material loading of 20 mg cm^–2^. For comparative analysis, a control group containing the same amount (0.5 wt %) of carbon additive and active material loading was also fabricated. It is noteworthy that with a polyvinylidene fluoride (PVDF) binder content below 3 wt %, the control electrodes did not form properly, necessitating the use of a 3 wt % binder. Additionally, the electrodes were compressed to a density of ∼3.0 g cm^–3^, the maximum achievable with our calendaring machine without inducing particle fracture.

**Figure 4:**
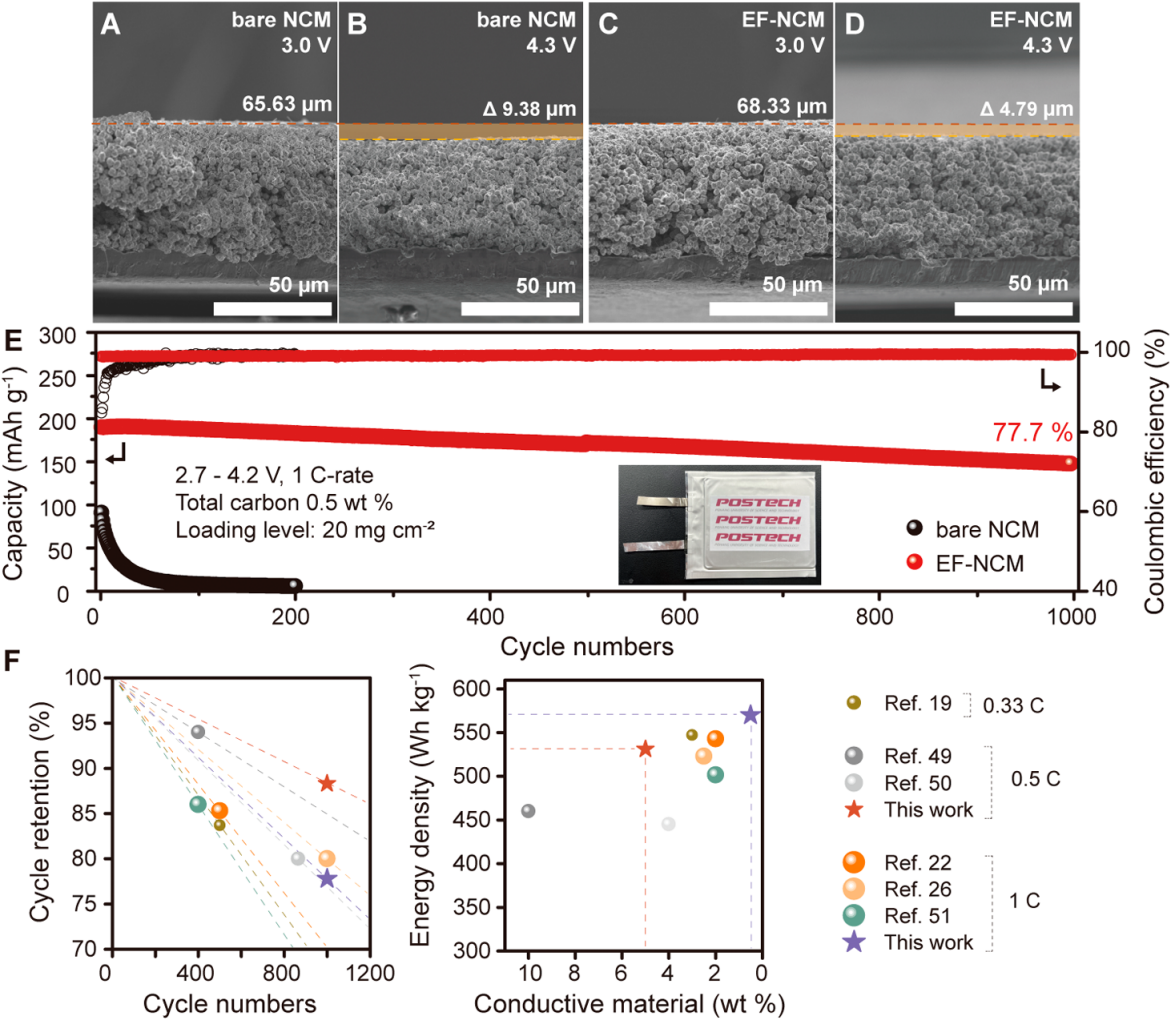
Electrode thickness tracking and comparison of electrochemical performance. (a) Bare NCM electrode at 3.0 V state, (b) bare NCM electrode at 4.3 V state, (c) EF-NCM at 3.0 V state, and (d) EF-NCM at 4.3 V state. (e) Cycle life test result of EF-NCM with 0.5 wt % conductive carbon and 20 mg cm^–2^ loading level. (The logo presented in Figure 4E is used with proper acknowledgment from the copyright holder.) (f) Comparison of energy density, ratio of conductive material, and cycle life of pouch cell consisting of nickel-rich cathode which have long-term cycle life (the plotted data are also provided in Tables S1 and S2). Gravimetric energy density (Wh kg^–1^) was calculated including the weight of the Al foil current collector (0.1240 g, 30.4 cm^2^). The thickness and density of the Al foil were 15 μm.^47−51^

Both the bare NCM (65.625 μm, Figure 4A) and EF-NCM (68.333 μm, Figure 4C) electrodes exhibited comparable thicknesses at 3.0 V after the formation cycle. However, upon charging to 4.3 V, the EF-NCM electrode displayed a thickness reduction of 4.79 μm (Figure 4D), while the bare NCM electrode exhibited a greater contraction of 9.38 μm (Figure 4B). This result demonstrates that the elastic framework significantly reduces electrode thickness variation. To further verify these thickness changes, *ex-situ* measurements using a micrometer were conducted (detailed information is provided in Figure S30). These results also highlight the role of EF-NCM in mitigating volume contraction through its mechanical resilience.

To validate the volume-controllable design in an *in situ* environment, *in situ* dilatometry analysis was conducted. During cycling, the bare NCM9055 cathode exhibited a thickness contraction of approximately 2.5 μm at 4.3 V (a 3.3% decrease in the thickness of the cathode slurry region), whereas the EF-NCM cathode demonstrated a significantly reduced contraction of nearly 50% less at 4.3 V (<1 μm, a 1.45% decrease in the thickness of the cathode slurry region). This observation aligns with the first charge data presented in Figure S26a and is further corroborated by Figure S31.

The pouch cell utilizing the EF-NCM electrode demonstrated remarkable long-term cycling stability, achieving 77.7% capacity retention after 1000 cycles at a 1 C rate. (Additionally, the cycle retention data at various rates are provided in Figures S32 and S33.) Initially, the bare NCM electrode exhibited reasonable charge and discharge capacities (Figure S34); however, it underwent rapid capacity degradation, approaching nearly zero capacity within 100 cycles (Figure 4E). This rapid decline in capacity is likely due to electrode contact issues exacerbated by the minimal use of carbon additives combined with commercial-level active material loading, rather than intrinsic material degradation.

**Scheme 1:**
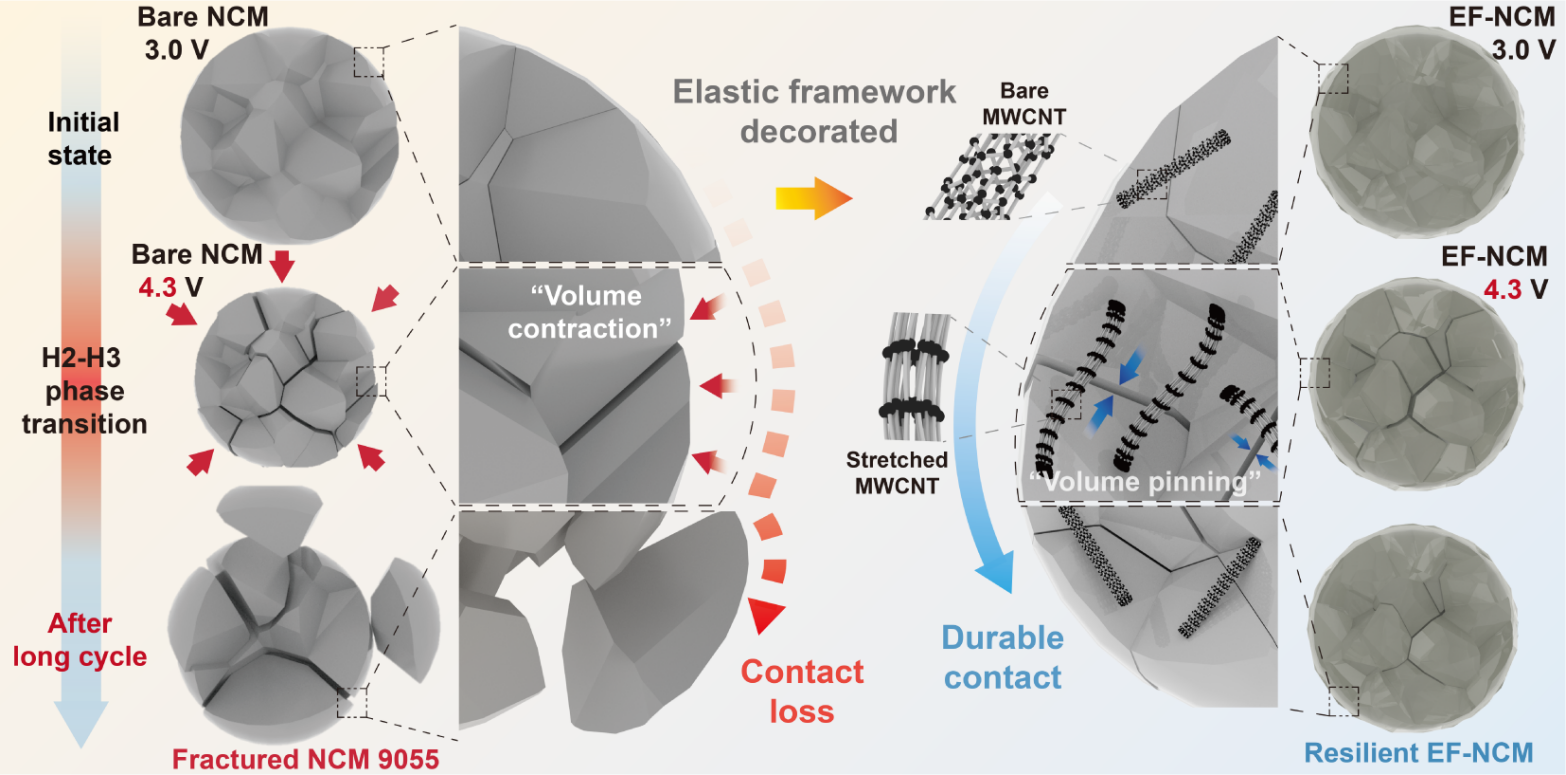
Schematic Illustration of Volume Pinning Strategies for Long Cycle LIBs

This advanced electrode design, featuring exceptionally low carbon content (<0.5 wt %) and high loading levels (>20 mg cm^–2^), enables unprecedented practical energy density at the electrode level (570 Wh kg^–1^), even when accounting for typically inactive components such as carbon additives and the current collector. As shown in Figure 4F, the performance of the EF-NCM electrode surpasses that of previously reported Ni-rich cathodes in terms of cycle life, reduction of inactive components, and resulting practical energy density. Conclusively, the EF-NCM effectively mitigates volume changes and contact loss (Scheme 1), achieving a commercial-level lifespan and active material loading.

## Conclusion

Addressing electrochemical instability caused by lattice volume changes is critical for high-energy-density electrode designs. To this end, our research introduces an elastic framework that provides a comprehensive solution to challenges ranging from the particle to the electrode level. Specifically, the MWCNT framework effectively absorbs mechanical energy during particle shrinkage and releases it during relaxation, thereby enhancing crack resilience and mitigating contact loss between electrode particles. Additionally, the elastic strain generated by the framework minimizes secondary particle volume contraction, significantly reducing thickness changes in the electrode architecture and improving contact stability, even during prolonged cycling. This mechanical resilience enables electrode designs with high active material loading (20 mg cm^–2^), exceptionally low carbon content (<0.5 wt %), and extended cycle life, achieving 77.7% capacity retention after 1000 cycles. This study not only presents a novel particle design strategy utilizing the elastic framework but also demonstrates how controlling mechanical resilience properties can overcome the challenges associated with high-energy-density electrodes and related applications that suffer from cyclical volume changes.

## Methods

### Materials

The multiwall CNT (JENOTUBE 6A) for preparing the EF-NCM was purchased from JEIO. Acetonitrile solvent (99.9%, HPLC plus grade), hexane (99%), and ethyl cellulose (viscosity 4 cP, 5% in toluene/ethanol 80:20) were purchased at Sigma-Aldrich. The LiNi_0.9_Mn_0.05_Co_0.05_O_2_ (NCM9055) precursor was synthesized via coprecipitation. NiSO_5_·6H_2_O, CoSO_4_·7H_2_O, and MnSO_4_·H_2_O precursor were mixed in an aqueous solution for 5 h to prepare the homogeneous solution. This solution was injected into the reaction chamber under controlled N_2_ gas. NaOH (Transition metal: NaOH ratio = 1:2) and NH_4_OH (TM: NH_4_OH = 1:1) solution were pumped into the reaction chamber. The temperature, pH, and stirring speed of the chamber were maintained at 50.5 °C, 11.3, and 1000 rpm during the reaction. The synthesized precursor was washed with D.I water and dried at 70 °C. The NCM9055 powder was prepared at O_2_ atmosphere flow furnace at 740 °C, 8 h.

### EF-NCM Preparation

A dispersion of multiwalled carbon nanotubes (MWCNTs) in ethyl cellulose (EC) with a ratio of 1:5 was prepared in acetonitrile solvent via tip sonication (VCX 750, Sonics) for 30 min, resulting in a well-dispersed MWCNT dispersion in the acetonitrile solution. Subsequently, to create a Pickering emulsion, hexane was introduced into the acetonitrile-based MWCNT dispersion at a ratio of 1:8. The size of the Pickering emulsion was designed to correspond with the size of the active material. In this emulsion, stabilized by the MWCNT, NCM9055 powder was inserted.^39^ The NCM9055 powder was mixed into the Pickering emulsion solution and subjected to fractional distillation to selectively remove the inner solvent, ensuring a homogeneous coating quality. The distillate powder was vacuum-filtered and dried in a 60 °C vacuum oven. To stably affix the MWCNT onto the active surface, EC was pyrolyzed at 240 °C for 1 h to secure the electronic conductivity induced by π–π interaction between EC residual and MWCNT and maximize the path of lithium-ion by removing the residual EC.^60^ During pyrolysis, EC transformed to amorphous carbon, forming a chemical bond between the MWCNT and the active powder. Subsequently, the EF-NCM powder was stored in an argon glovebox.

### Material Characterization

The surface morphology and crack propagation patterns were observed through Field emission SEM (FE-SEM, S-4800, HITACHI). Atomic resolution images were obtained via Field Emission Transition Electron Microscopy (FE-TEM, FEM-2100F, JEOL) at 200 kV, 50 μA condition. Synchrotron X-ray Diffraction (XRD) analysis was conducted at Pohang Light Source-II (PLS-II) 9B High-resolution powder diffraction (HRPD) beamline. This XRD analysis was operated at 10° to 130°, 0.01° step, 1 s exposure condition. X-ray nano tomography (TXM) image was prepared at 7C X-ray Nano Imaging (XNI) beamline at PLS-II. The field of view and pixel size were selected to 55 μm and 45 nm (2 bins was used in this work). To obtain each tomography image, 900 projection images were collected with 0.4 s exposure time and reconstructed through the filtered back algorithm method of Octopus software (TESCAN). *In-situ* XRD analysis was conducted at 6D UNIST-PAL beamline at PLS-II. The cross-section samples for SEM analysis were processed through cross-section polisher (SM-09010, JEOL). X-ray absorption (XAS) analysis was performed at 7D XAFS beamline using the Si (111) double crystal monochromator. Ni foil was used for energy calibration, and all XAS data was collected with transmission mode. Athena program was used for processing the XAS data. Thermo-Gravimetry analysis (TGA, SDT Q600, TA Instruments) was conducted at 10 °C min^–1^ heating rate. Raman analysis (FEX, NOST) was prepared with conditions such as step 0.83 Raman shift and 531 nm^–1^ wavelength laser sources. Time of flight mass spectrometry (TOF-SIMS, M6, IONTOFGmbH) analysis was utilized to acquire surface information on 1–3 nm. The Fullprof program was used for Whole-pattern matching of HRPD analysis. Nanoindentation analysis (FISCHERSCOPE HM2000, Helmut Fischer GmbH) was conducted on individual cathode particles dispersed on a glass slide. During the test, a total load of 20 mN was applied over a duration of 50 s. A flat punch indenter tip was used for the measurements. *In-situ* dilatometry analysis (DS800S, Magnescale) was performed to monitor the volume variation of the cathode electrode. To minimize the volume change of the anode, a Lithium Titanium Oxide (LTO) anode, known as a zero-strain material, was used.^61^ For the cathode, electrodes with a high loading (∼21 mg cm^–2^) were fabricated, consisting of 96.5 wt % cathode material, 0.5 wt % carbon, and 3 wt % PVDF binder to enable clear observation of thickness variation. The thickness variation was calculated excluding the thickness of the Al foil. The full cell was assembled with an N/P ratio of 1.1. Cycling was conducted at a 0.2 C-rate between 1.5 and 2.8 V, considering the high redox potential of LTO during the phase transition from Li_4_Ti_5_O_12_ to Li_7_Ti_5_O_12_ (∼1.5 V). X-ray photoelectron spectroscopy (XPS, K-Alpha^+^, Thermo Fisher Scientific) was performed using a 200 μm X-ray source and calibrated based on the C 1s peak at 284.5 eV.

### Computational Methods

The finite element analyses were performed using COMSOL Multiphysics V6.2. To investigate the separation of primary particles within secondary particles, Central Voronoi Tessellation (CVT) was employed to create the geometry of the secondary particles with a radius of 2.5 μm. A Multiphysics simulation was utilized to simulate the volume contraction and internal Li diffusion in the bare NCM particle, incorporating both solid mechanics and transport of diluted species. This Multiphysics approach enabled the simulation of mechanical and chemical changes simultaneously. The total strain tensor, *ε*_*ij*_, of the NCM particle is expressed as,
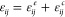
where,  is the elastic strain tensor, and  is the compositional strain tensor.^62^ The elastic strain tensor, , is defined by the stress–strain relationship given as,
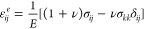
where *E* is the elastic modulus of NCM, which is 135 GPa, *v* is Poisson’s ratio, which has value of 0.3, and *δ*_*ij*_ is the Kronecker delta.^63^ The compositional strain tensor, which describes the volume change of the particle, is expressed as,
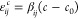
where *β*_*ij*_ is the expansion coefficient, *c* represents the Li concentration, and *c*_0_ is the initial Li concentration of 31097.64 mol/m^3^.^64^ This tensor reflects the changes in lattice parameters in varying Li content.

The diffusion of Li inside the NCM particle is governed by the mass conservation equation, represented by Fick’s second law of diffusion.^65^ The Fick’s second law of diffusion is expressed as,
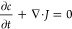
where *J* is the Li flux to the active material particle. Li flux, *J*, is defined by Fick’s first law of diffusion,
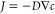
where *D* is the Li diffusivity inside the active material particle, the value of which is indicated in Figure S21c.^66^ The MWCNT framework was also generated using CVT, and beam physics was applied to model its mechanical properties. The MWCNT framework was simulated as a beam structure with a 5 nm radius, a Young’s modulus of 1800 GPa, and a Poisson’s ratio of 0.07. To attach the MWCNT beam on the surface of the NCM, a solid-beam connection Multiphysics module was employed. The equation for attaching MWCNT on the NCM surface is given as,
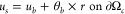
where *u*_*s*_ and *u*_*b*_ are the displacement vector of the surface of the solid and the beam, *θ*_*b*_ represents the rotation vector of the beam, *r* is the position vector from the center line of the beam, and ∂Ω_c_ denotes the boundary of the solid.^67^ This equation models the mechanical interaction between the beam and the surface of the particle.

To simulate the grain boundary separation, thin elastic layer node under solid mechanics physics was applied. It was assumed that primary particles are connected by imaginary springs to each other, so generating tensile stress would produce microcracks within the particle. The separation due to spring elongation at the thin elastic layer is expressed as,
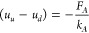
where (*u*_*u*_*– u*_*d*_) represents the displacement of primary particles, *F*_*A*_ is the applied force per unit area, and *k*_*A*_ is the stiffness of the imaginary spring, with a value of 10^9^ N/(m·m^2^).^68^ This model accounts for the failure that occurs at the grain boundaries due to mechanical stress.

### Electrode Preparation and Electrochemical Characterization

The cathode slurry, which was fabricated with *N*-Methyl-2-Pyrolidone (NMP), active materials, super C65 (TIMCAL), and PVDF binder (KF 1120, KUREHA), was prepared for cell test. This mixture was cast on Al foil and dried at 120 °C convection oven. The electrodes were prepared with a high carbon ratio (active material: conductive material: binder = 90:5:5) and low carbon ratio (active material: conductive material: binder = 96.5:0.5:3). The loading level of the electrode was controlled to 5 mg cm^–2^ or 20 mg cm^–2^. 2032-coin cell kit, glass fiber (Whatman), Li metal (Honzo), and 1 M LiPF_6_ in ethylene carbonate (EC): dimethyl carbonate (DMC): ethyl methyl carbonate (EMC) = 3:4:3 (v/v/v) + 3 wt % VC electrolyte was used for coin cell fabrication.

The pouch cell was fabricated with graphite anode at 1.2 of N/P ratio. The coin cell test was conducted between 3.0 and 4.3 V with battery cycler WBCS 3000 (WONATECH). Pouch cell test was cycled between 2.7 and 4.2 V. The GITT analysis was prepared with 10 min electrochemical stimulation process and 30 min rest condition.

## Supporting Information Available

Schematic illustration of supporting materials for proving the resilience effect of elastic framework; SEM images; GCD; TGA; Raman; TEM; HRPD; *In situ* XRD; statistical volume tracking result; simulated geometry of cathode particles; GITT; lattice parameters contracted from HRPD; simulated parameters; particle cross section images; Statistical analysis of internal microcrack, simulated result of finite element method, battery cycle life test; XANES and EXAFS; rate properties test; nano indenter test; *in situ* dilatometry test; XPS analysis; table of pouch cell design and cycle life (PDF)
Nano CT movie of bare NCM cathode after 200 cycles (MP4)
Nano CT movie of EF-NCM cathode after 200 cycles (MP4)
