# Supplementary material for: Enhancing Mechanical Resilience in Li-Ion Battery Cathodes with Nanoscale Elastic Framework Coatings
Source: ACS Nano. 2025 Jan 3;19(1):1588–99. doi: 10.1021/acsnano.4c14980 (PMC11753261; doi:10.1021/acsnano.4c14980)
Supplement: Supplementary file 1 — nn4c14980_si_001.pdf [file nn4c14980_si_001.pdf]

Enhancing Mechanical Resilience in Li-Ion Battery Cathodes with Nanoscale Elastic Framework Coatings

Jong-Heon Lim[a†], Jaehyun Kim[b†], Jiwoong Oh[a], Jaesub Kwon[c], Kyoung Eun Lee[a], Youngsu Lee[a], Seongeun Park[a], Jun Lim[d], Dongwook Shin[e], Changshin Jo[a,f], Yong-Tae Kim[c], Janghyuk Moon[b], Mark C. Hersam[g,h,i]*, and Kyu-Young Park[a,c]**

[a]Graduate Institute of Ferrous & Eco Materials Technology (GIFT), Pohang University of Science and Technology University, Pohang, 37666, Republic of Korea

[b]Department of Energy Systems Engineering, Chung-Ang University, Seoul, 06974, Republic of Korea

[c]Department of Materials Science and Engineering (MSE), Pohang University of Science and Technology University, Pohang, 37673, Republic of Korea

[d]Pohang Accelerator Laboratory (PAL), Pohang University of Science and Technology, Pohang, 37673, Republic of Korea

[e]Materials Development Group, Samsung SDI, Samsung Future Technology Campus, 130 Samsung-ro, Yeongtong-gu, Suwon, Gyeonggi, 16678, Republic of Korea

[f] Department of Chemical Engineering, Pohang University of Science and Technology University, Pohang, 37673, Republic of Korea

[g]Department of Materials Science and Engineering, Northwestern University, Evanston, IL 60208, USA

[h]Department of Chemistry, Northwestern University, Evanston, IL 60208, USA

[i]Department of Electrical and Computer Engineering, Northwestern University, Evanston, IL 60208, USA

*Email: kypark0922@postech.ac.kr; m-hersam@northwestern.edu; jhmoon84@cau.ac.kr

[†]J.H. Lim, and J. kim contributed equally to this work.

KEYWORDS: lithium-ion battery, surface modification, carbon nanotube, elastic framework, mechanical resilience

Table S1. Electrode design for long cycle life and high powder density pouch cell.

Cathode material	Active loading level (mg cm^{-2})	Conductive agent (wt %)	Electrolyte	Cell configuration	Voltage (V)	Cycle retention	Ref.
NCM76	18.5	2.0 wt % (Super P)	1.2 M $LiPF_6$ EC: EMC=3:7 (v/v) + 2wt % VC	Pouch cell	2.6 - 4.3 V	83.7% 500 cycle	(*19*)
NCMo95	9-10	3.0 wt % (Carbon black)	1.2 M $LiPF_6$ EC: EMC=3:7 (v/v) + 2wt % VC	Pouch cell	3.0 - 4.2 V	85.3%,1C 500cycle	(*22*)
$LiNi_{0.94}Co_{0.06}O_2$	11.5	2.5 wt % (Carbon black)	1.0 M LiFSI – 0.5 M $LiPF_6$/EMC + 3% VC	Pouch cell	2.5 - 4.2 V	80%, 1C 1000cycle	(*26*)
$LiNi_{0.8}Co_{0.1}Mn_{0.1}O_2$	20.0	10.0 wt % (Carbon black)	1.0 M $LiPF_6$ EC: DEC=3:7 (v/v) +3 wt % FEC	Pouch cell	2.8 - 4.2 V	94%, 0.5C 400 cycle	(*49*)
$LiNi_{0.8}Co_{0.1}Mn_{0.1}O_2$	12.4	4 wt % (Super C65)	1.0 M $LiPF_6$ EC: EMC=3:7(v/v)	Pouch cell	2.5 - 4.3 V	80%, 0.5C, 865 cycle	(*50*)
$LiNi_{0.8}Co_{0.1}Mn_{0.1}O_2$	12.0	2 wt % (Super P)	1.3 M $LiPF_6$ EC: EMC: DEC=3:6:1 (v/v/v) +1wt % VC + 0.5 wt % 1,3-propane sultone	Pouch cell	2.8 – 4.2 V	86%, 1C 400 cycle	(*51*)
**$LiNi_{0.9}Co_{0.05}Mn_{0.05}O_2$**	**20**	**4.5 wt % Super C 65 + 0.5 wt % elastic framework (MWCNT)**	**1.0 M $LiPF_6$ EC: DMC: EMC=3:4:3(v/v/v) +3 wt % VC**	**Pouch cell**	**2.7 – 4.2 V**	**88.4%, 0.5C 1000 cycle**	**This work**
**$LiNi_{0.9}Co_{0.05}Mn_{0.05}O_2$**	**20**	**0.5 wt % elastic framework (MWCNT)**	**1.0 M $LiPF_6$ EC: DMC: EMC=3:4:3(v/v/v) +3 wt % VC**	**Pouch cell**	**2.7 – 4.2 V**	**77.7%, 1C 1000 cycle**	**This work**

* Reference numbers of this table was cited by main manuscript.

Table S2. Detail information for energy density calculations

Reference number	Loading level (mg cm^{-2})	Initial capacity (mAh g^{-1})	Carbon ratio	Average voltage (V)	Active mass ratio (%)	Active mass (mg)	Energy density with Al foil (Wh/kg)
(*19*)	18.5	200	2.0 wt % (Super P)	3.45	96	562.4	542.74
(*22*)	9	235	3.0 wt % (Carbon black)	3.6	94	273.6	547.23
(*26*)	11.5	235	2.5 wt % (Carbon black)	3.35	90	349.6	523.02
(*49*)	20	195	10.0 wt % (Carbon black)	3.55	80	608	459.99
(*50*)	12	200	4 wt % (Super C65)	3.5	96	364.8	501.53
(*51*)	12.4	192	2 wt % (Super P)	3.35	92	376.96	445.27
**This work**	**20**	**206**	**4.5 wt % Super C 65 + 0.5 wt % elastic framework (MWCNT)**	**3.45**	**90**	**608**	**569.65**
**This work**	**20**	**206**	**0.5 wt % elastic framework (MWCNT)**	**3.45**	**96.5**	**608**	**531.28**

* Reference numbers of this table was cited by main manuscript.

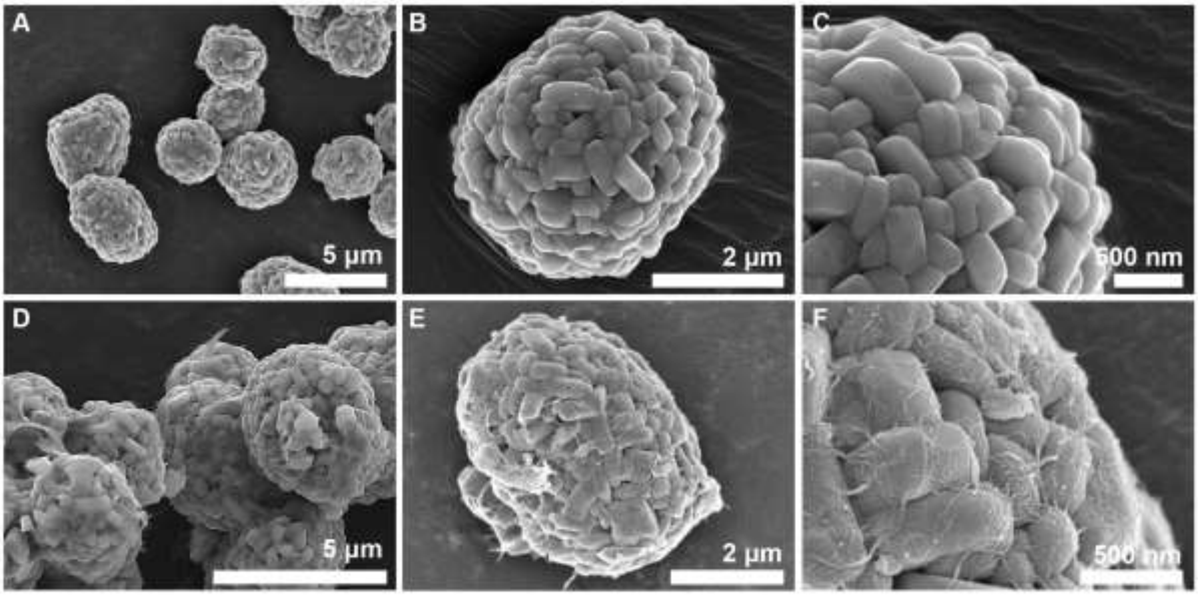

Figure S1. FE-SEM analysis result of (A-C) bare NCM and (D-F) EF-NCM.

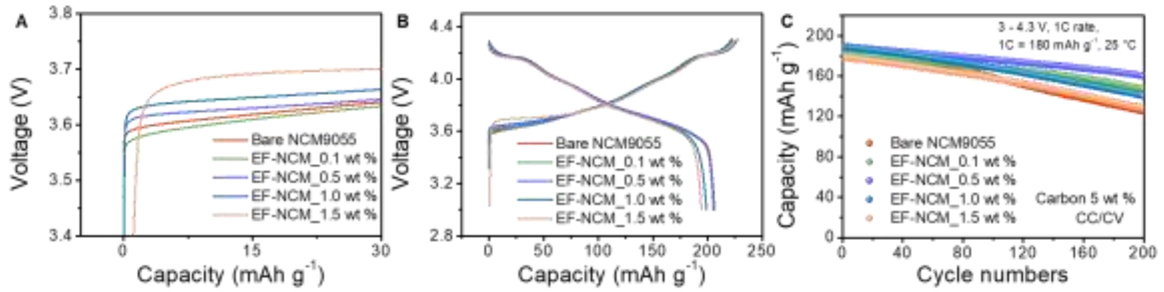

Figure. S2 Charge discharge curve (A, B) and cycle performance graph (C) of bare NCM and 0.1, 0.5, 1.0, 1.5wt % CNT decorated EF-NCM.

The optimization process focused on electrochemical performance, considering (i) initial capacity and (ii) cycle retention. In terms of initial capacity, EF-NCM with 0.1-0.5 wt % CNT coatings exhibited values comparable to bare NCM9055, while higher coating ratios (>1.0 wt %) resulted in reduced capacities (~8-10 mAh g^{-1} lower). After 200 cycles, the highest capacity retention (84.8%) was achieved with 0.5 wt % CNT-coated EF-NCM. Lower coating ratios, such as 0.1 wt %, exhibited slightly reduced retention (81%) due to insufficient CNT coverage. FEM analysis identified the cause of capacity degradation as the limited mechanical resilience of the coarsely coated elastic framework. Specifically, FEM simulations demonstrated that EF-NCM with 0.1 wt % MWCNT coating exhibited significantly larger surface displacement (~30 nm) and higher elastic strain (3.05%), resulting in an increased total strain (2.78%) compared to EF-NCM with 0.5 wt % MWCNT coating, which exhibited a surface displacement of only ~5 nm (Figure S3). Based on these findings, 0.5 wt % was identified as the optimal CNT coating ratio, balancing high capacity retention with comparable initial capacity.

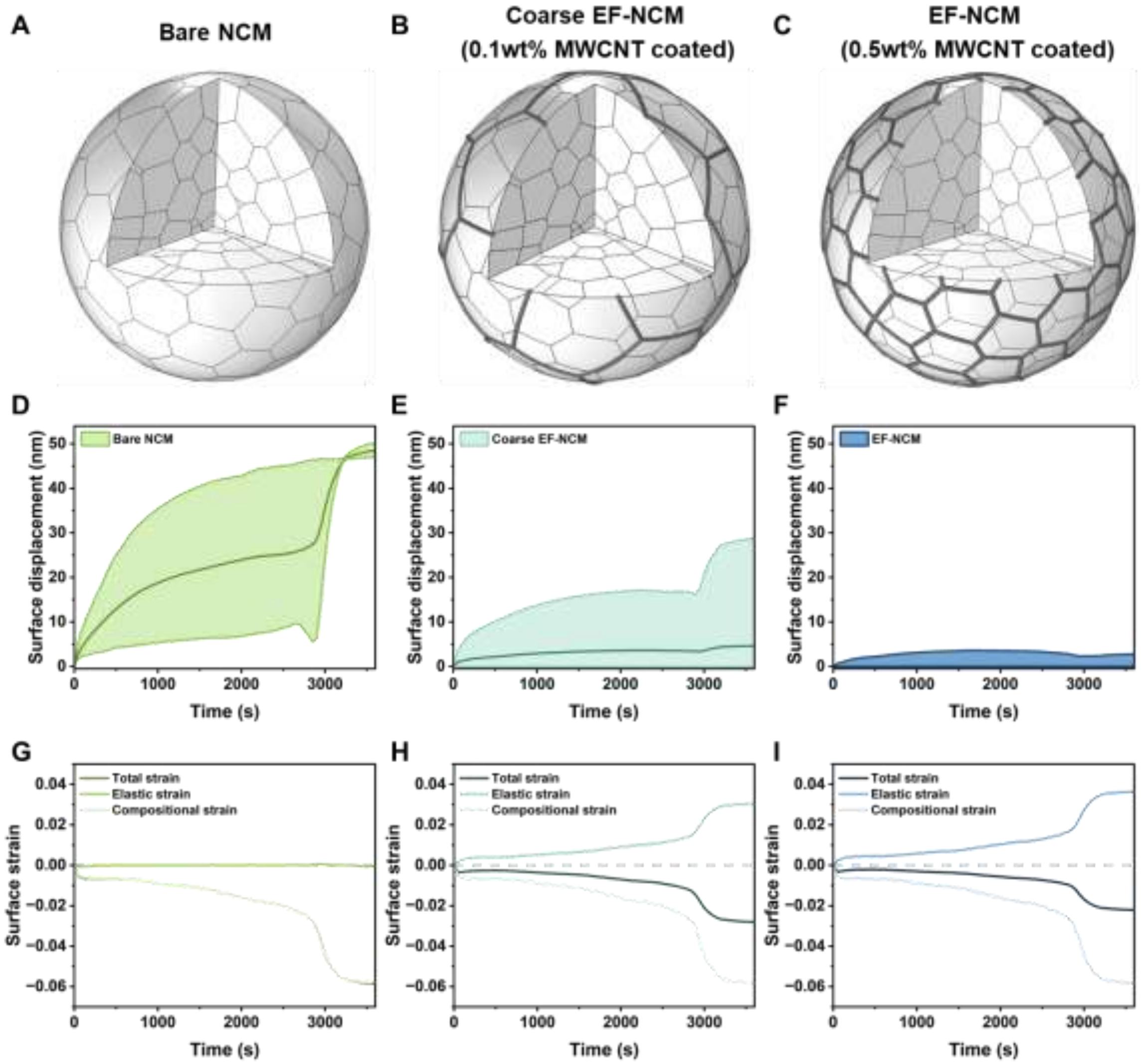

Figure S3. Simulated geometry of (A) bare NCM, (B) 0.1wt % MWCNT coated EF-NCM, and (C) 0.5wt % CNT coated EF-NCM. Displacements and surface strain analysis of (D, G) bare NCM, (E, H) coarse EF-NCM, and (F, I) EF-NCM observed during charge process.

Three types of NCM secondary particle geometries: bare NCM, 0.1wt % MWCNT coated EF-NCM, and 0.5wt % CNT coated EF-NCM were designed, as illustrated in Figure S3A-C, to estimate the effect of insufficient CNT coverage. The length of CNT coating on the surface of the coarsely coated EF-NCM was set to be 3.26 times the value of the particle surface area ($\frac{Total\ length\ of\ CNT}{Surface\ area} = 3.26\ \mu m^{-1}$), while for 0.5 wt % CNT coated EF-NCM, it was set to 6.77 ($\frac{Total\ length\ of\ CNT}{Surface\ area} = 6.77\ \mu m^{-1}$). The mechanical behavior of the three types of secondary particles during charging processes was simulated, revealing differences in volume

changes. As indicated in the manuscript, the bare NCM exhibited a surface displacement of approximately 50 nm in its radius, whereas the 0.5 wt % CNT coated EF-NCM showed a maximum surface displacement of about 5 nm (Figure S3D, F) However, the 0.1 wt % CNT coated EF-NCM exhibited a significantly larger maximum surface displacement of approximately 30 nm, which is markedly increased compared to 0.5 wt % CNT coated EF-NCM due to the insufficient CNT framework (Figure S3E). While the strain components of the 0.1 wt % CNT coated EF-NCM were shown to be similar to those of the 0.5 wt % CNT coated EF-NCM, differences were observed in elastic strain and total strain values (Figure S3G, I). Specifically, the 0.1 wt % CNT coating resulted in 3.05% in elastic strain, leading to the total strain of 2.78% contraction in the NCM particle (Figure S3H).

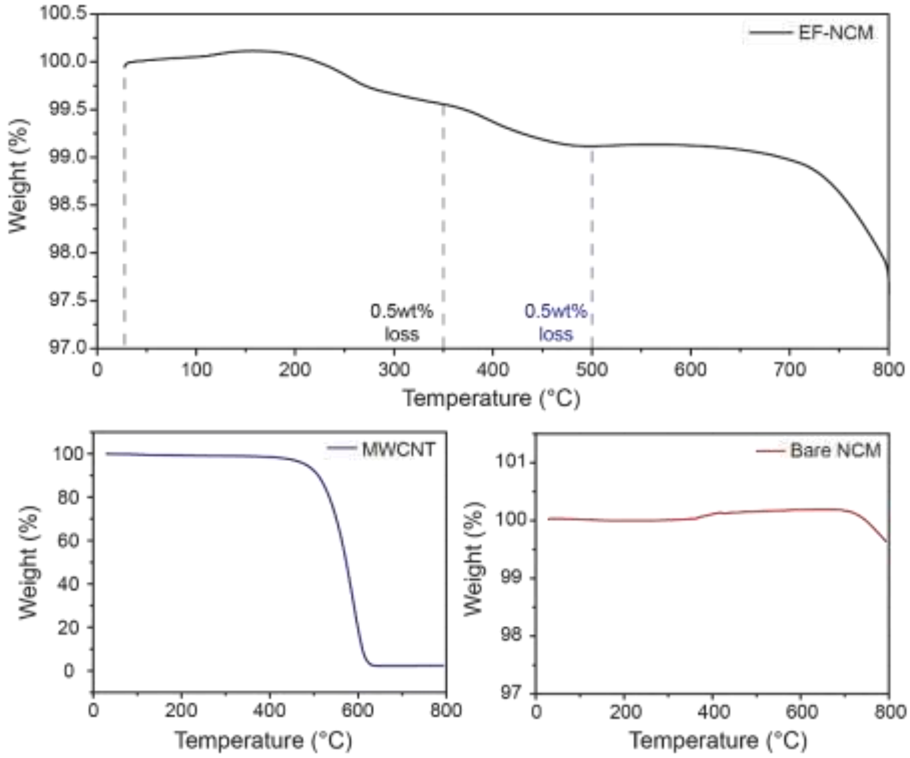

Figure S4. TGA analysis result of EF-NCM

Under ambient air conditions, ethyl-cellulose decomposition was observed below 350 °C, followed by weight loss attributed to MWCNT oxidation occurring between 450 °C and 600 °C. Thermal degradation beyond 750 °C was attributed to the NCM9055 cathode.[1,2]

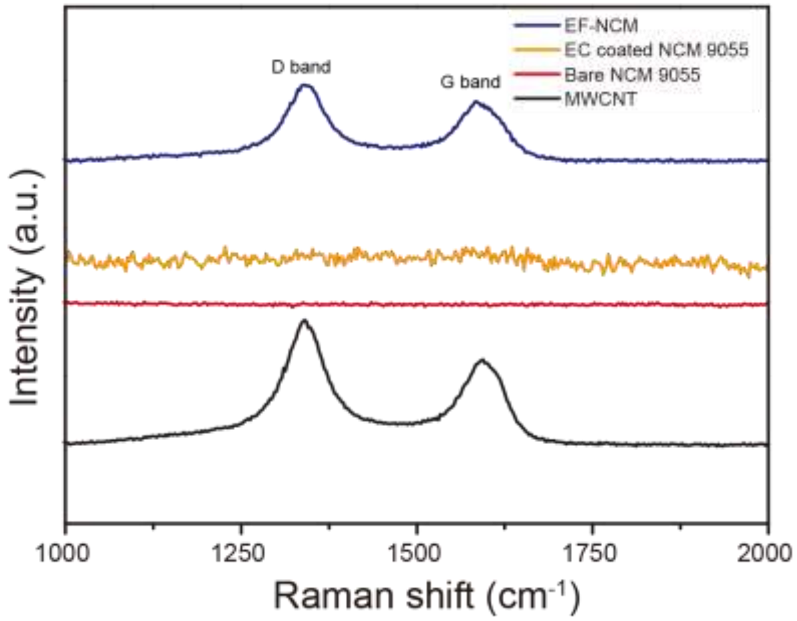

Figure S5. Raman analysis result of EF-NCM, EC coated NCM9055, bare NCM9055, and MWCNT.

Raman spectroscopy is one of the tools for elucidating the characteristics of the CNT through the distinctive D and G band peaks, representing the defect and SP^2 bonding structure, respectively.[3,4] The clear D and G band peaks were observed in the Raman spectroscopy results of MWCNT and EF-NCM, presented at 1338 cm^{-1} and 1594 cm^{-1}. On the other hand, the original NCM9055 and EC-coated NCM9055 exhibited nearly undetectable intensity within the 1000~2000 cm^{-1} range, indicating that the distinct D and G bands observed in the EF-NCM spectra originated from the MWCNT.

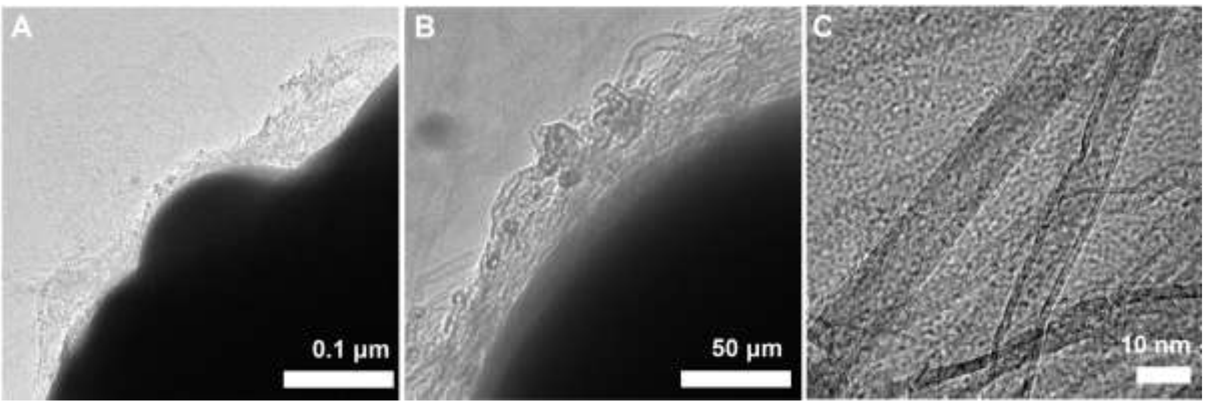

Figure S6. TEM analysis for atomic scale observation; (A, B) MWCNT-based elastic framework on EF-NCM, and (C) shape of MWCNT.

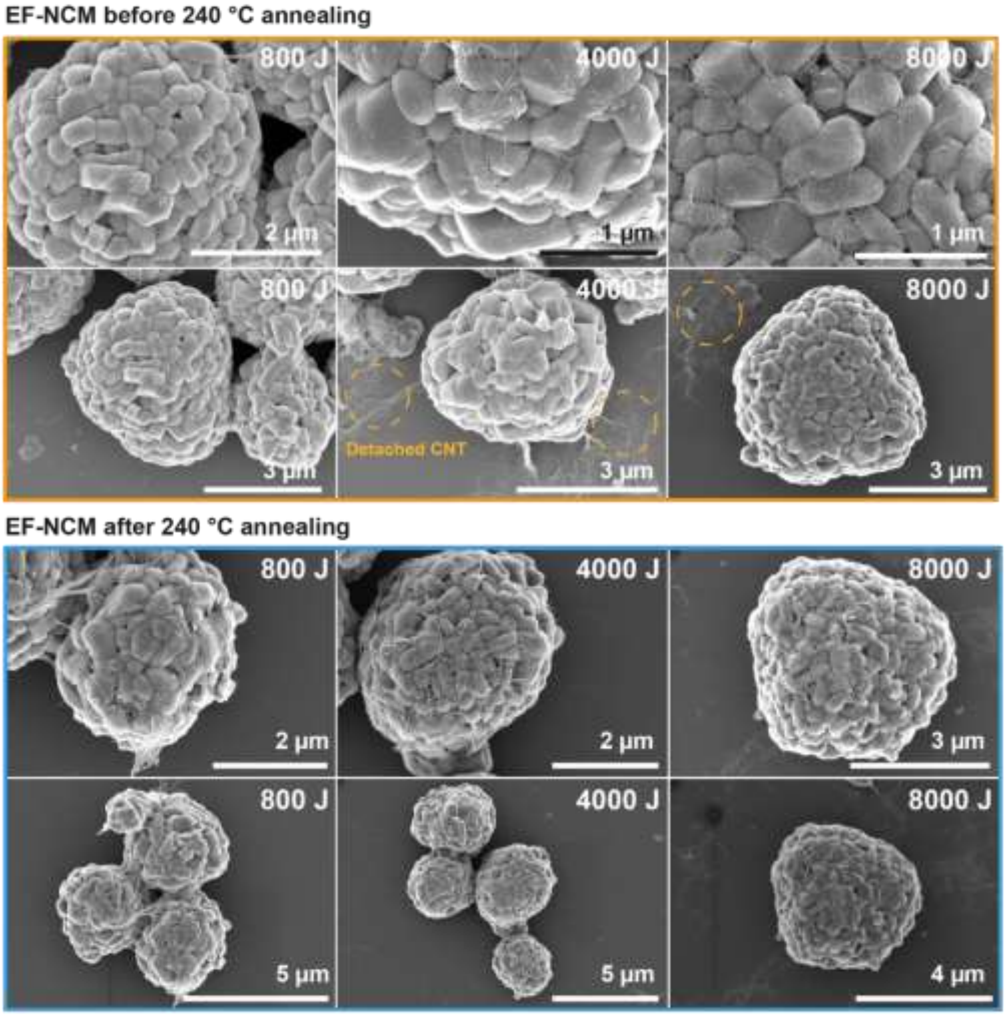

Figure S7. Sonicated EF-NCM before and after 240 °C annealing for observing CNT binding quality. This sample was prepared at the tip sonicator at 15 ml acetonitrile solution with 0.05 g of EF-NCM powder.

The detachment test was prepared to prove the stable adhesion of CNT particles on EF-NCM. The sonicated EF-NCM powder was prepared with 1, 5, 10 min prove sonication (800, 4000, 8000 J separately) at acetonitrile solvents which are used in the coating process. Even after the detachment process, three kinds of EF-NCM samples, which conduced 240 °C annealing, had stable elastic framework layer on the active surface. However, EF-NCM before 240 °C annealing showed remarkably detached CNT particles after applied energy over 4000 J. Through this result, we demonstrate the stubborn coating quality of EF-NCM.

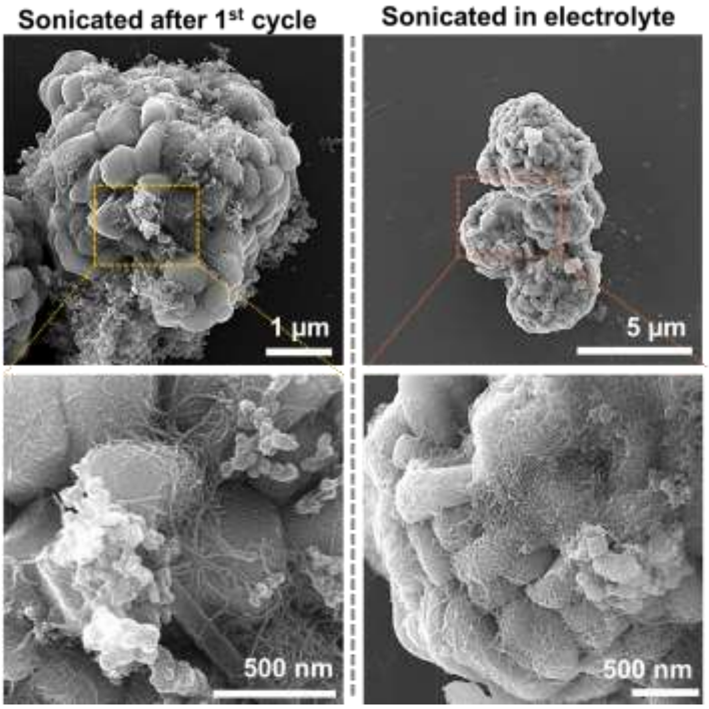

Figure S8. SEM analysis result of EF-NCM after attachment test

A sonication test was conducted to evaluate the attachment capability of the elastic framework. After applying 4000 J per gram to EF-NCM using the method described in Figure S7, the elastic framework was observed to remain well-attached in both the first-cycled cathode and the cathode sonicated in the electrolyte (as described in the Experimental Section).

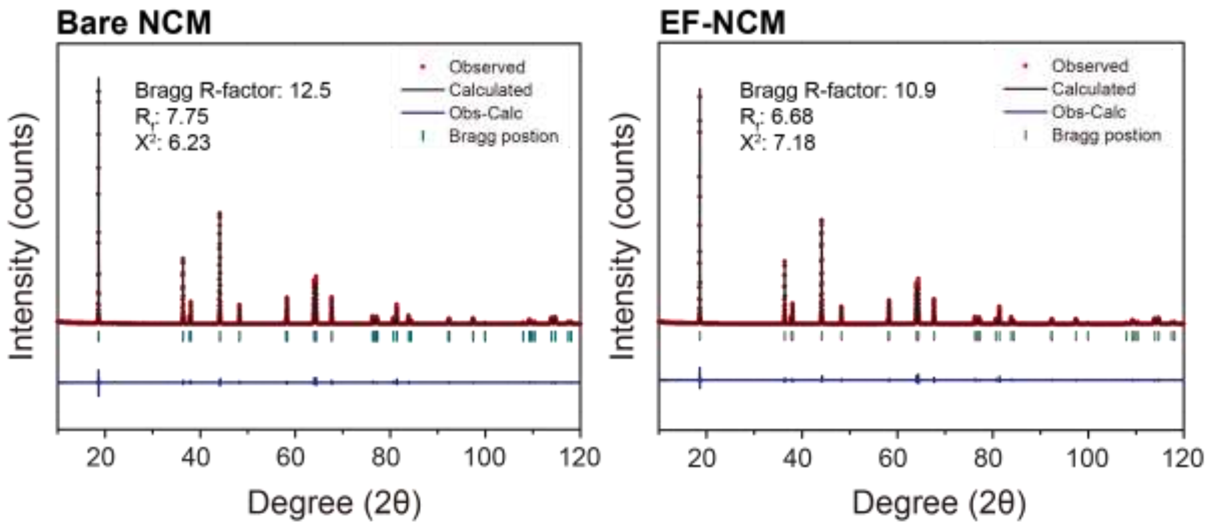

Lattice information

Sample	a (Å)	b (Å)	c (Å)	volume (Å^3)	R_p	R_{wp}	R_{exp}
Bare NCM	2.87211 (1)	2.87211 (1)	14.19297 (5)	101.392	12.8	16.2	5.83
EF-NCM	2.87272 (1)	2.87272 (1)	14.19492 (5)	101.45	12.2	15.5	5.78

Figure S9. Whole-pattern matching result of bare NCM and EF-NCM

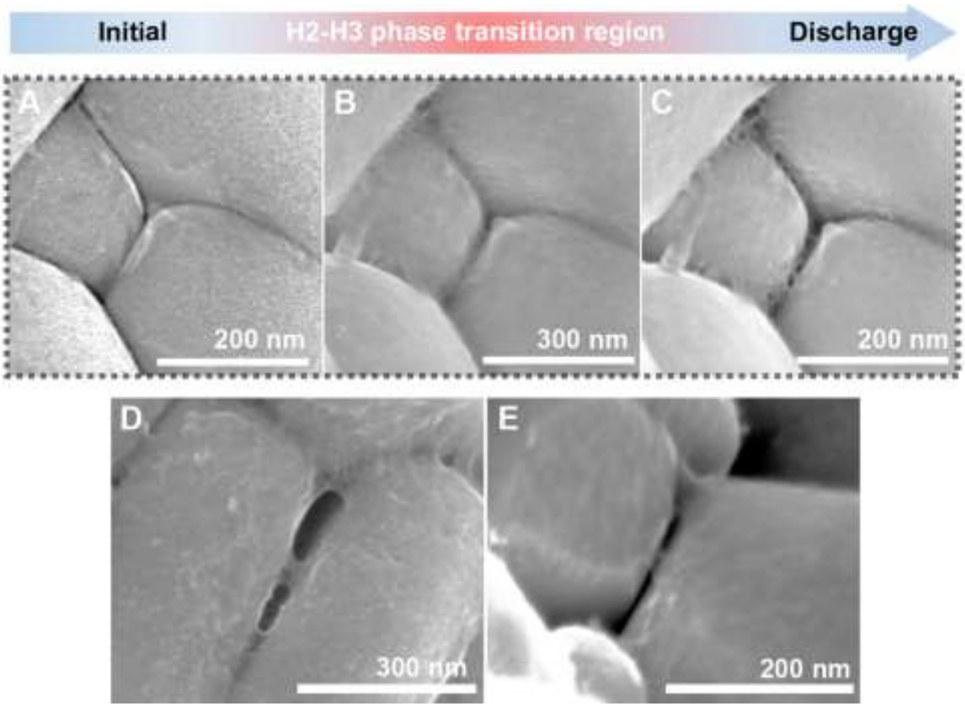

Figure S10. Crack generation behavior of bare NCM before cycle (A), after 1st charge (B), and 1st discharge (C-E).

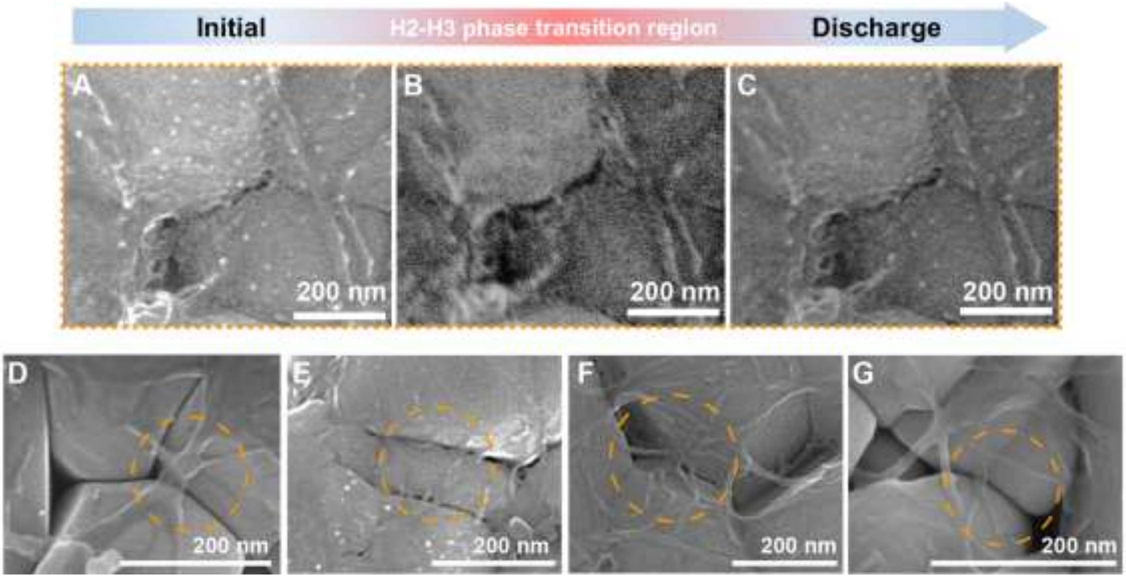

Figure S11. Crack generation behavior of EF-NCM before cycle (A), after 1st charge (B), and 1st discharge (C-G).

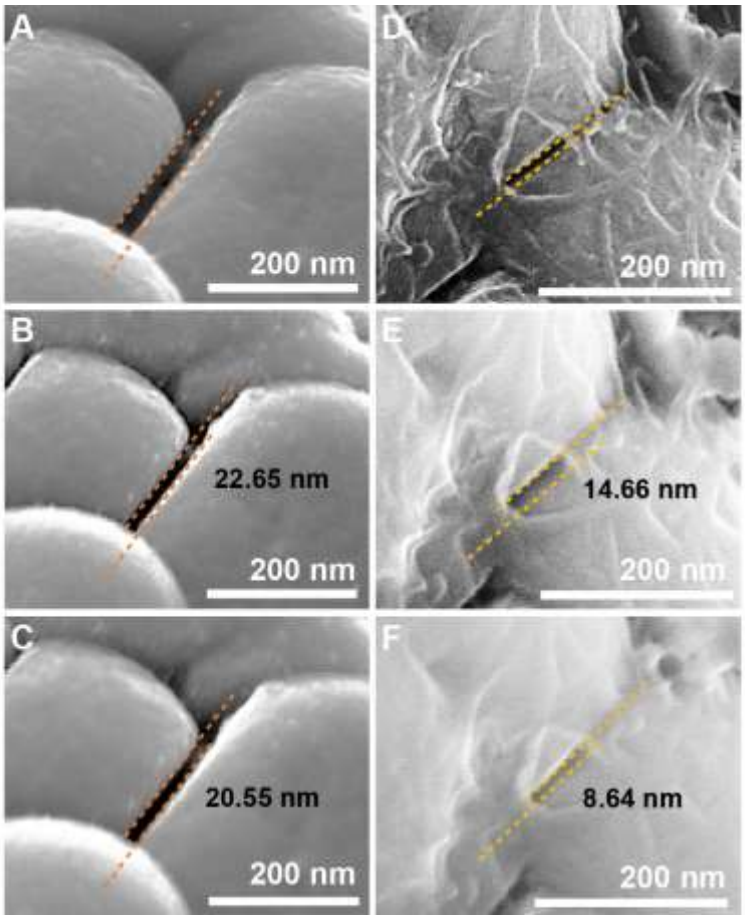

Figure S12. Crack generation behavior of bare NCM and EF-NCM at 2nd charge-discharge state; (A) bare NCM 2nd initial state, (B) bare NCM 2nd charge, (C) bare NCM 2nd discharge, (D) EF-NCM 2nd initial state, (E) EF-NCM 2nd charge, (F) EF-NCM 2nd discharge.

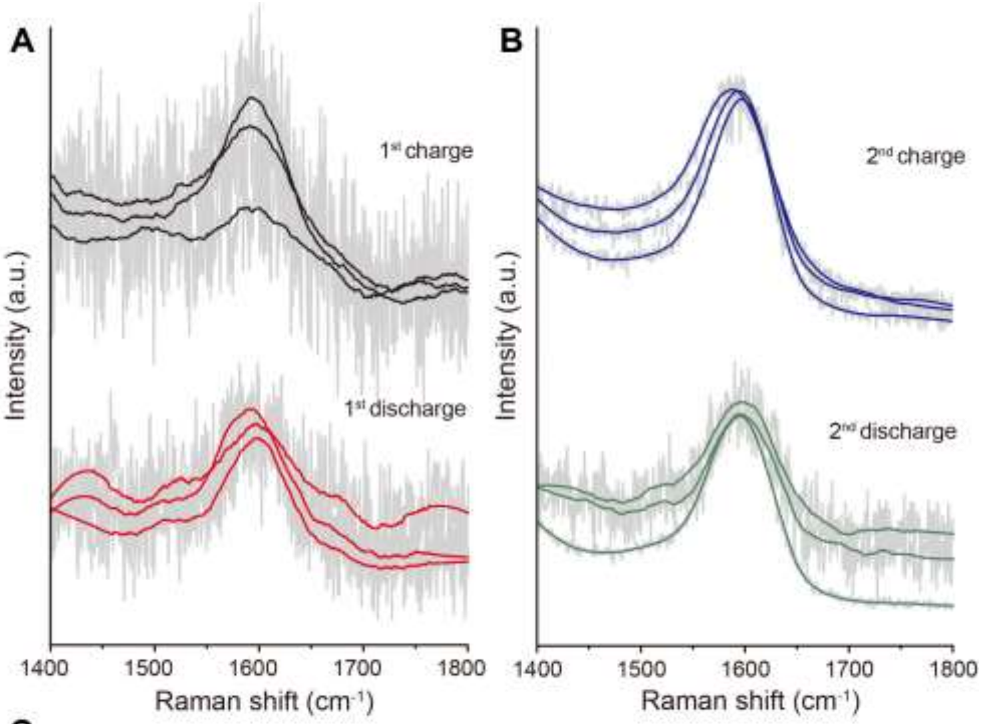

C

	Initial	1st charge	1st discharge	2nd charge	2nd discharge
**Raman shift (cm^{-1})**	1595.64	1591.93	1596.12	1593.33	1596.68
**Strain rate (%)**	0	1.0912	-0.1422	0.6794	-0.3069

Figure S13. G-band shift result of EF-NCM at specific charge-discharge state; (A) 1st charge, 1st discharge, (B) 2nd charge, 2nd discharge, (C) statistical value table of Raman shift and strain variation.

To observe the subtle Raman peak shift, the EF-NCM electrode was designed using EF-NCM powder and PVDF, without conductive carbon. The electrode was prepared for each charge-discharge state, with Raman analysis conducted at 0.88 cm^{-1} intervals to detect delicate shifts in the Raman spectra. The resulting Raman spectra were normalized and smoothed to enhance the primary features by noise removal.

The strain ratio of the MWCNT was calculated using the equation:

$$K = v\varepsilon + v_o$$

K denotes the shifted peak wavenumber (cm^{-1}), v represents the wavenumber/peak shift (cm^{-1}/%) and v_o represents the original peak wavenumber. The factor v, which reflected the average strain rate degree of MWCNT from 0° to 180° was calculated to 3.4 cm^{-1}/%.[5]

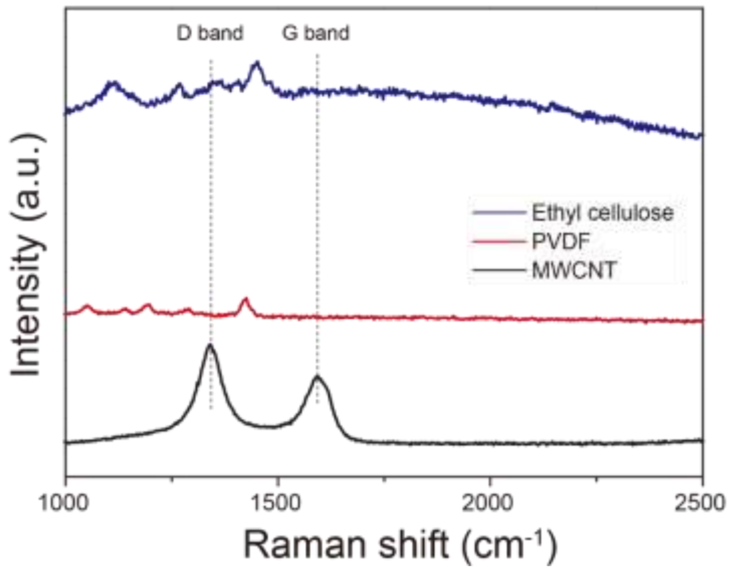

Figure S14. Raman analysis result of MWCNT, PVDF, and EC.

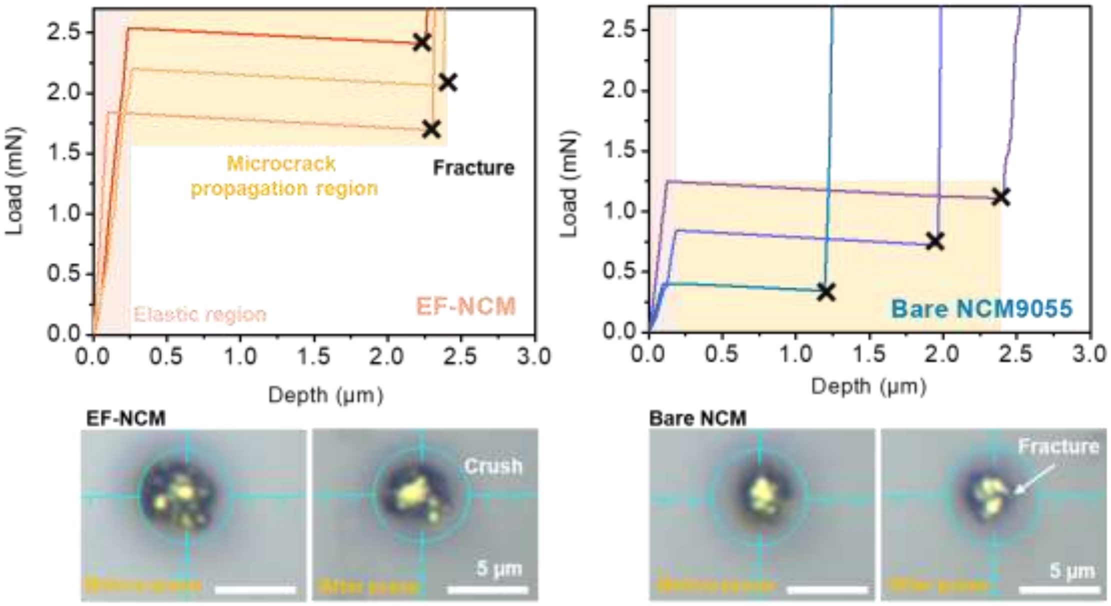

Figure S15. Load displacement curve of bare NCM and EF-NCM

A nanoindenter was employed to measure the mechanical properties of the materials. The results indicate that the secondary active materials exhibit an elastic region and a microcrack propagation region prior to fracture.[6] Notably, CNT-decorated NCM (EF-NCM) demonstrated a higher critical fracture load (~2 ± 0.5 mN) compared to bare NCM9055 (~1 ± 0.5 mN), indicating that EF-NCM possesses significantly greater initial stiffness. Furthermore, the fracture morphology of bare NCM particles displayed a fractured shape, while EF-NCM particles exhibited a crushed morphology, likely due to the additional reinforcement effect provided by the elastic framework.

According to the Griffith equation for brittle fracture, factors such as Young's modulus, flaw size, and fracture energy contribute to mechanical strength.[7] Among these, fracture energy, which is directly related to the energy required to create new surfaces, can be significantly influenced by the Pickering emulsion coating process.[8] As reported by Hsieh et al., frameworks incorporating MWCNTs exhibit enhanced fracture energy due to the CNTs'

ability to absorb mechanical energy.[9] This increase in fracture energy provided by the MWCNT framework accounts for the higher stiffness observed in EF-NCM.

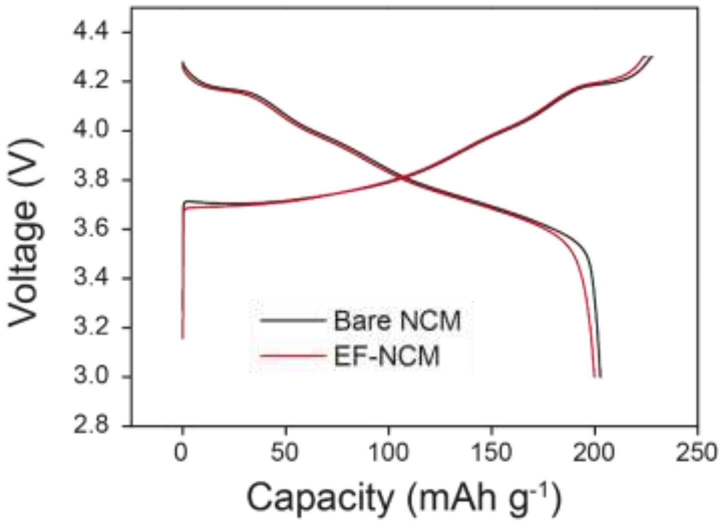

Figure S16. GCD graph of electrode consisted with 20 wt % of conductive carbon, without calendaring process**.**

The bare NCM electrode for particle volume tracking was fabricated with 60:20:20 ratios of active material: conductive material: binder, and EF-NCM electrode was fabricated with 60: 0.5:19.5:20 ratios of active material: decorated MWCNT: conductive material: binder to form the electrochemically well-connected electrode. The calendaring process was excluded for omitting the mechanical damage. The charge-discharge protocol was conducted with CC/CV mode for guiding equal SOC state. As a result, the electrodes fabricated with 20 wt % conductive material exhibited 1st discharge capacity of 200 mAh g^{-1}, almost similar to the electrode with the calendaring process containing 5 wt % conductive material.

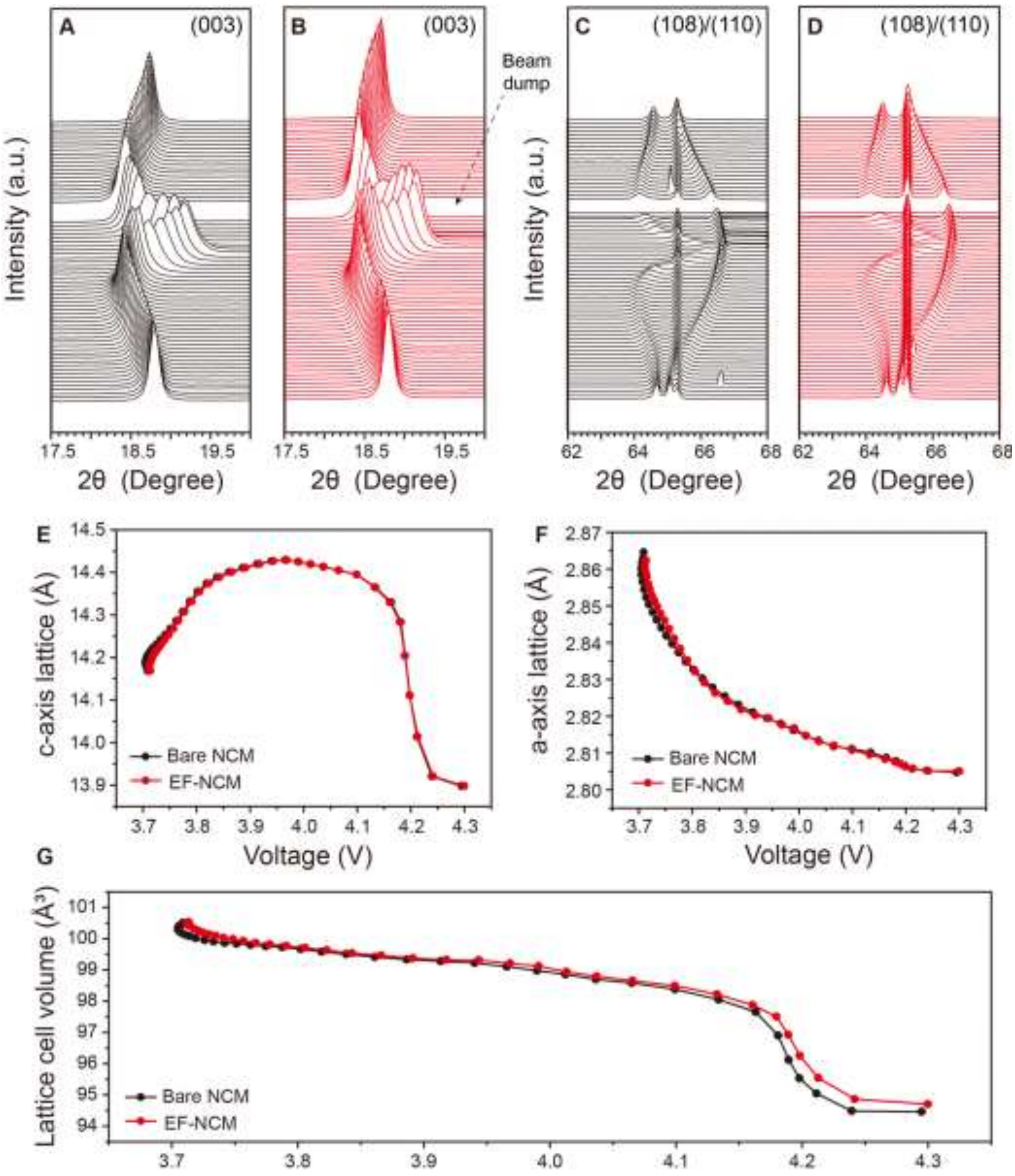

H

Sample	Bare NCM		EF-NCM	
	Volume	Deviation	Volume	Deviation
**Initial volume**	100.556	0.004	100.64	0.004
**Final volume**	94.746	0.009	94.704	0.006

Figure S17. *In-situ* XRD result during electrochemical stimulation and extracted lattice parameter of bare NCM and EF-NCM; (A) (003) peak of bare NCM, (B) (003) peak of EF-NCM, (C) (108)/(110) peak of bare NCM, (D) (108)/(110) peak of EF-NCM, (E) *c*-lattice parameter, (F) *a* -lattice parameter, (G) lattice volume parameter. (H) lattice volume deviation of bare and EF-NCM.

The lattice information of bare NCM and EF-NCM was obtained through *in-situ* XRD analysis. A significant contraction in the *c*-lattice parameter was observed above 4.1 V,

consistent with the behavior expected during the H2-H3 phase transition region (Figure S17E). Additionally, the a-lattice parameter (Figure S17F) exhibited a consistent volume contraction during electrochemical charging. Comparison of the *a* and *c*-lattice parameters revealed similar contraction rates in both bare and EF-NCM materials. the deviation of lattice volume variation was calculated through this equation.

$$\delta(x \pm y) = \sqrt{\delta x^2 + \delta y^2}$$

The calculated deviation was 0.0098 at bare NCM and 0.0072 at EF-NCM. Consequently, the lattice cell volume showed analogous contraction behavior due to the similar variation in lattice values.

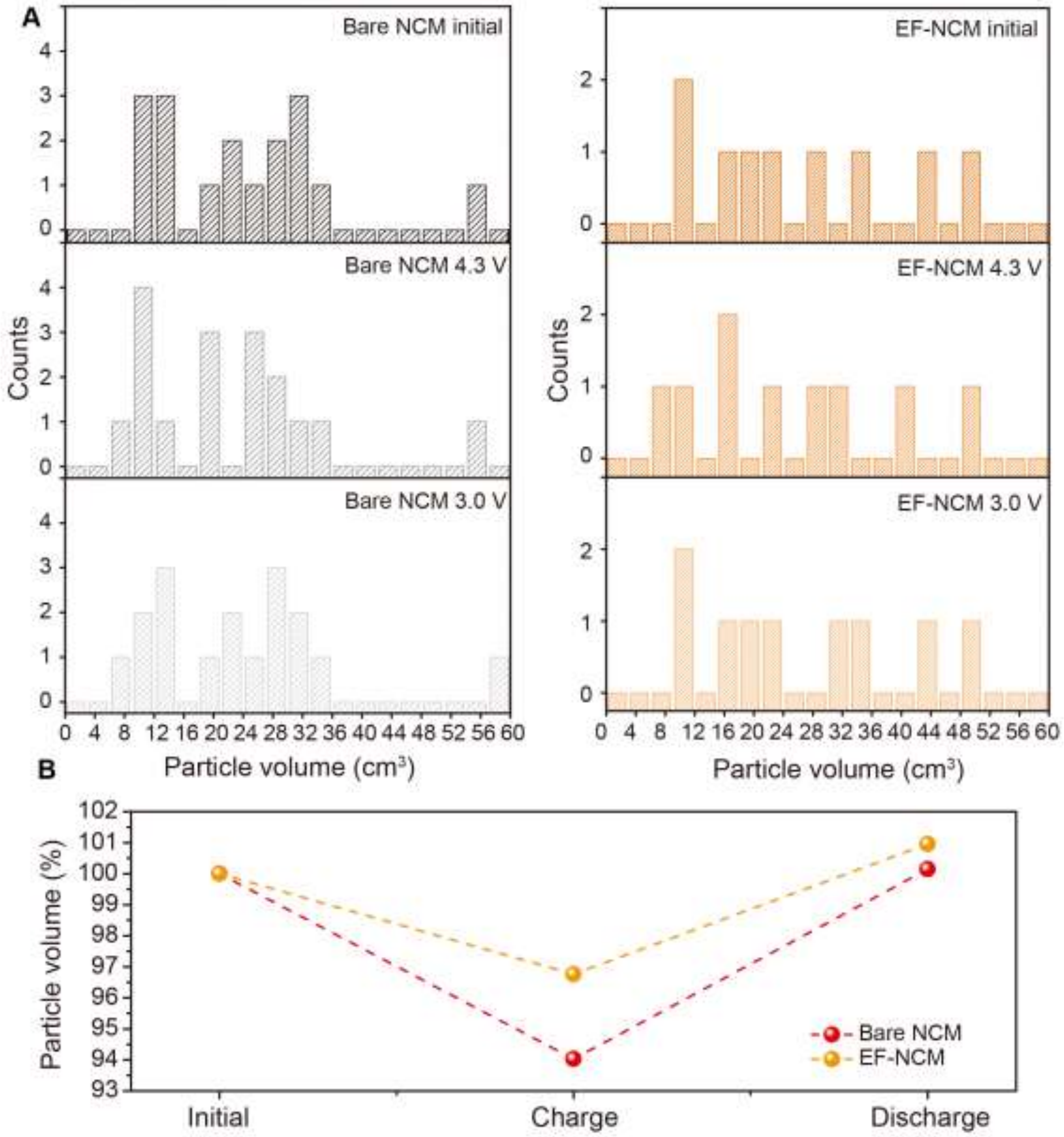

C

Variation	Bare NCM			EF-NCM		
	Initial	Charge	Discharge	Initial	Charge	Discharge
**Diameter (μm)**	3.508	3.439	3.516	3.576	3.526	3.584
**Volume (μm³)**	24.420	22.962	24.456	26.047	25.203	26.295
**Percent (%)**	100	94.028	100.147	100	96.760	100.953

Figure S18. (A) Volume tracking result of identical particles of bare NCM and EF-NCM. (B) Statistical particle volume tracking result of bare NCM and EF-NCM. (C) Table of statistically calculated volume tracking results.

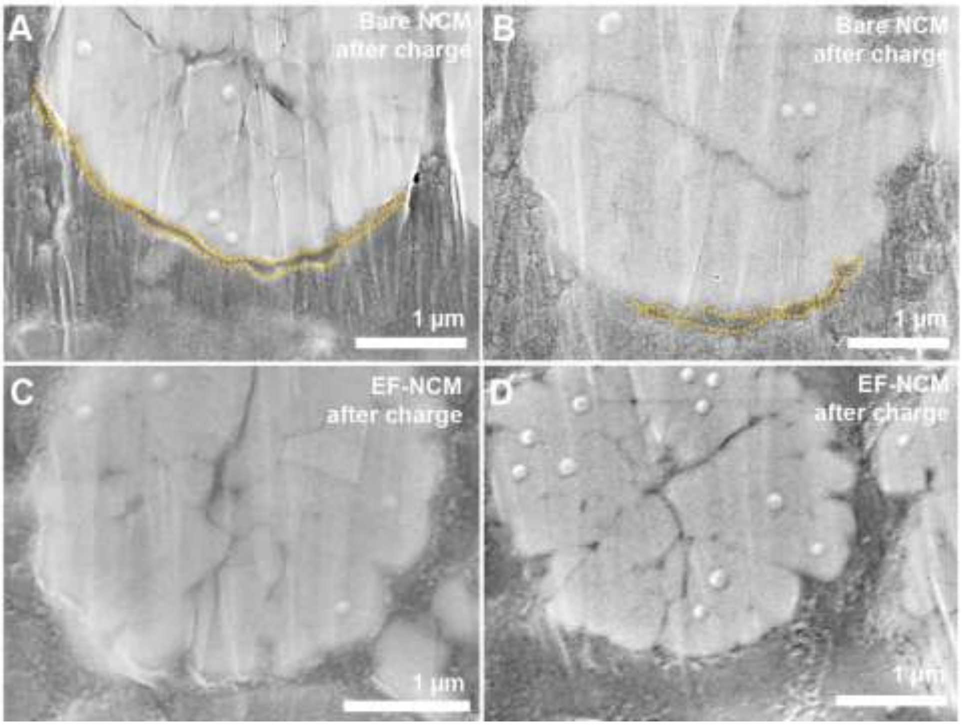

Figure S19. Cross-section SEM image of 4.3 V charged (A, B) bare NCM electrode and (C, D) EF-NCM electrode for observing contact properties of the particle to carbon domain.

To observe the contact properties between cathode particles and the conductive matrix, cross-sectional FE-SEM images were prepared using an Ar-ion polisher at 1st cycle. At the 4.3 V charge state, the bare NCM particles were separated from the conductive matrix. In contrast, EF-NCM presented well-contact properties with the conductive carbon domain. It is estimated that the elastic framework effectively prevents particle volume shrinkage, resulting in improved contact properties.

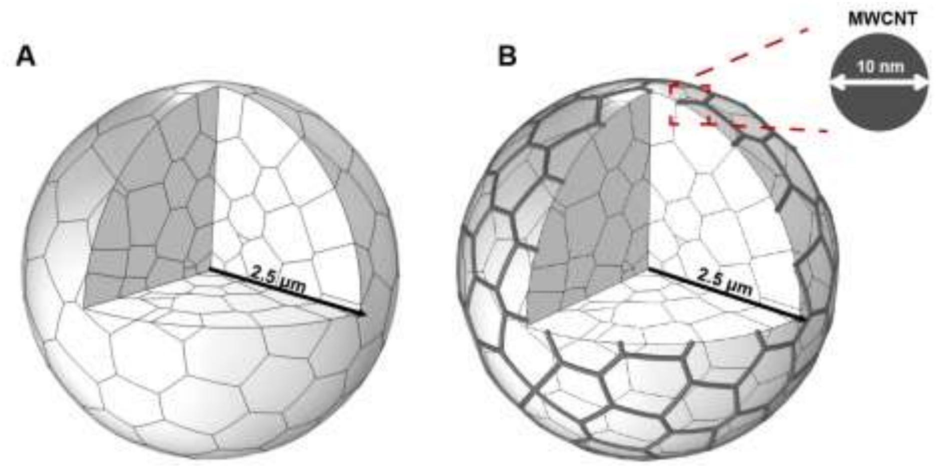

Figure S20. Simulated geometry of (A) bare NCM and (B) EF-NCM. The grey lines indicate the MWCNT elastic framework.

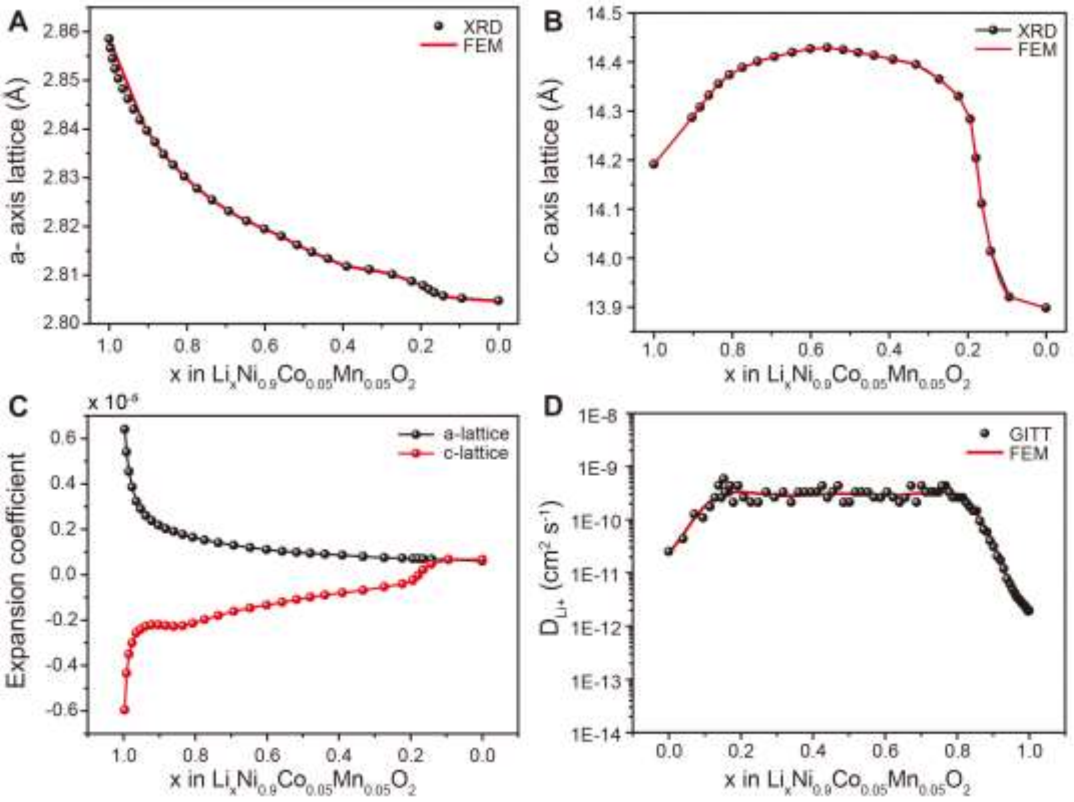

Figure S21. XRD measurements and simulated parameters of (A) *a*-axis and (B) *c*-axis. (C) Simulated lattice expansion coefficients of each lattice. (D) GITT measurement and simulated diffusion coefficient of NCM9055.

Table S3. Simulated parameters of bare NCM and EF-NCM

Symbol	Value	Description	Ref
E_{NCM}	$135\ GPa$	Elastic modulus of NCM9055	
E_{MWCNT}	$1800\ GPa$	Elastic modulus of MWCNT	
v_{NCM}	0.3	Poisson's ratio of NCM9055	
v_{MWCNT}	0.07	Poisson's ratio of MWCNT	(10)
$c_{max,NCM}$	$31097.64\ mol/m^3$	Stoichiometric Li concentration of NCM9055	
r_{NCM}	$2.5\ \mu m$	Particle Radius of NCM9055	
r_{MWCNT}	$5\ nm$	Radius of MWCNT	
k_A	$10^9\ N/(m \cdot m^2)$	Spring constant between NCM primary particles	

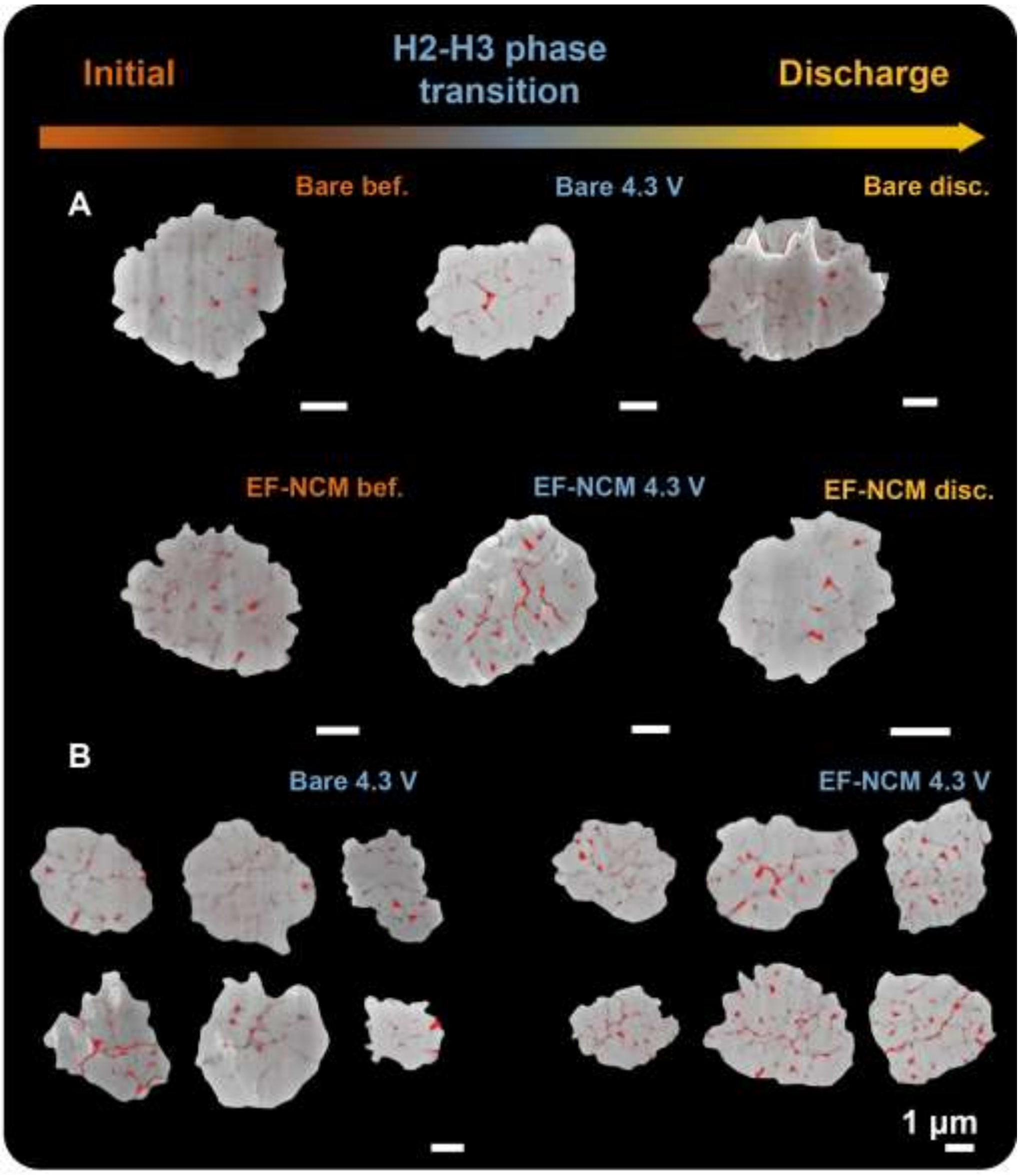

Figure S22. Cross-section SEM images during the electrochemical stimulation; (A) cross-section image of bare NCM and EF-NCM at initial, 4.3 V charged, and 3.0 V discharged, (B) collection cross-section images of bare NCM and EF-NCM at 4.3 V charged state.

To investigate the generation behavior of inner structural generation during the charging process, cross-sectional samples were prepared utilizing an Ar-ion polisher, and SEM images of polished particles were analyzed using ImageJ software. The internal structures of bare NCM and EF-NCM exhibited identical shapes, with inner space regions measuring 1.76% and 1.38%, respectively. Following a charge of 4.3 V, EF-NCM displayed a significant increase in inner space due to the volume pinning effect of the elastic framework. In contrast,

bare NCM showed a relatively smaller increase in inner space. These results were presented in the inner crack regions in bare NCM and EF-NCM, quantified at 2.66% and 4.47%, respectively. However, these generated cracks were found to be reversibly restored to their original shape after the 1st discharge, especially with the exterior elastic framework. Before cycling, the volume areas of bare NCM and EF-NCM were 1.76% and 1.38%, respectively.

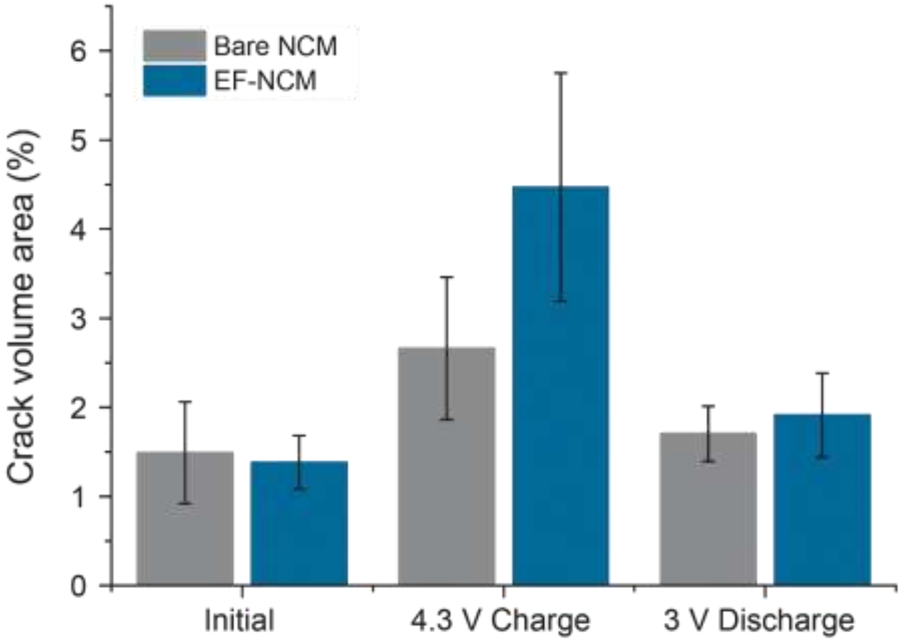

Figure S23. Statistical analysis of internal microcrack generation area at bare NCM and EF-NCM.

Statistical analysis of the crack formation in the bulk region is presented in Figure S23. This difference was believed that elastic framework on active surface facilitated the volume pinning when particle contraction, resulted in unforeseen internal space generation.

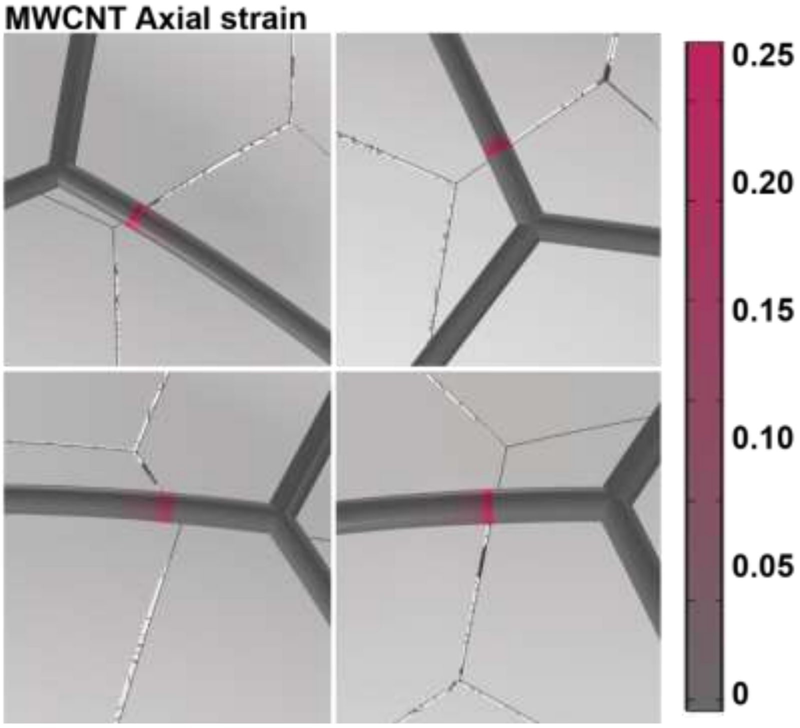

Figure S24. Axial strain distributions in MWCNT strands.

Due to the diameter of MWCNT, represented by the gray line, being prepared at 10 nm, the length of the axis in the upper image was calculated to be 130 nm. The scale of the sidebar denotes the elongated length relative to the original length and is expressed as a dimensionless value.

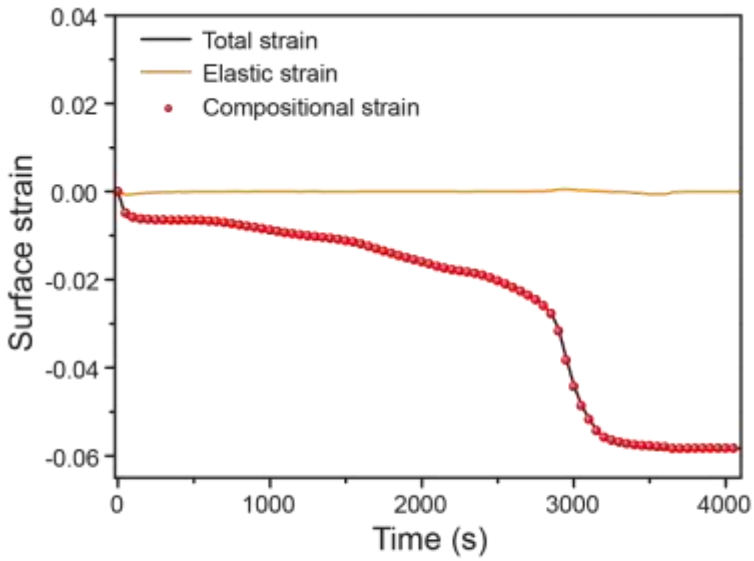

Figure S25. Strain analysis of bare NCM during the charge process via finite element method.

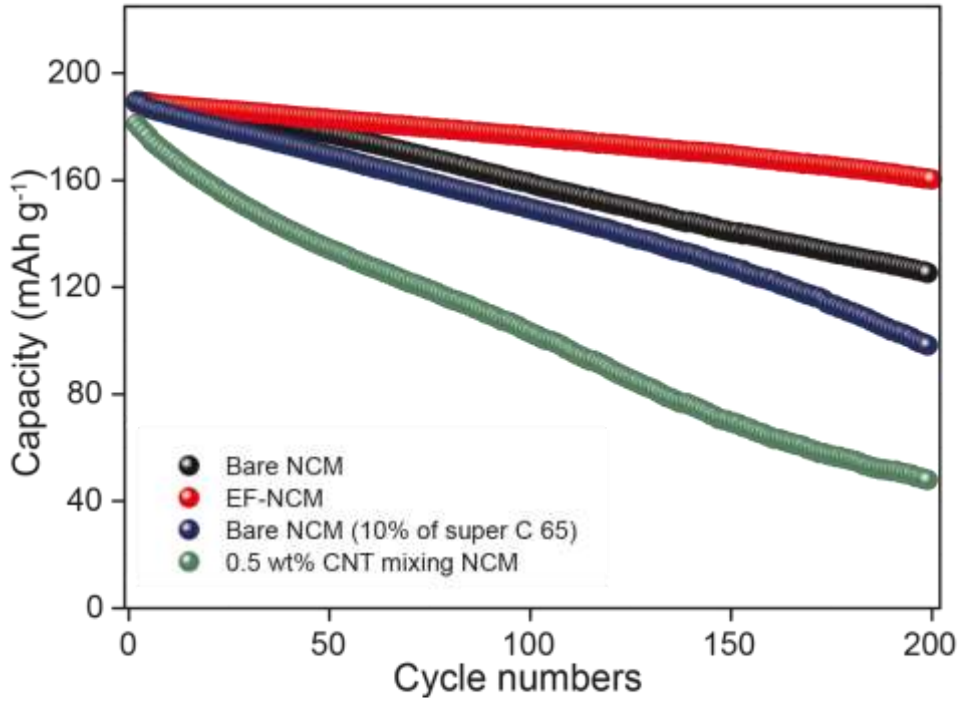

Figure S26. Coin cell test at 1C rate to prove the relationship between cycle life and volume hardening effect of elastic framework coated on EF-NCM.

The electrochemical performance of bare NCM and NCM cathodes with various conductive materials was tested to evaluate cycle life. The electrodes were fabricated with an active material ratio of 90:5:5, except for the electrode mixed with 10 wt % Super C65. The EF-NCM, which benefits from mechanical reinforcement and an efficient electron percolation path, demonstrated improved performance compared to bare NCM. Electrodes with a 10% carbon ratio exhibited rapid capacity fading relative to the 5% carbon-based NCM electrode, likely due to increased lithium-ion tortuosity.[11] The 0.5 wt % CNT-mixed electrode showed significantly decreased cycle retention, attributed to deteriorated electron percolation paths caused by CNT agglomeration during the fabrication process and compromised mechanical properties after cycling.[12]

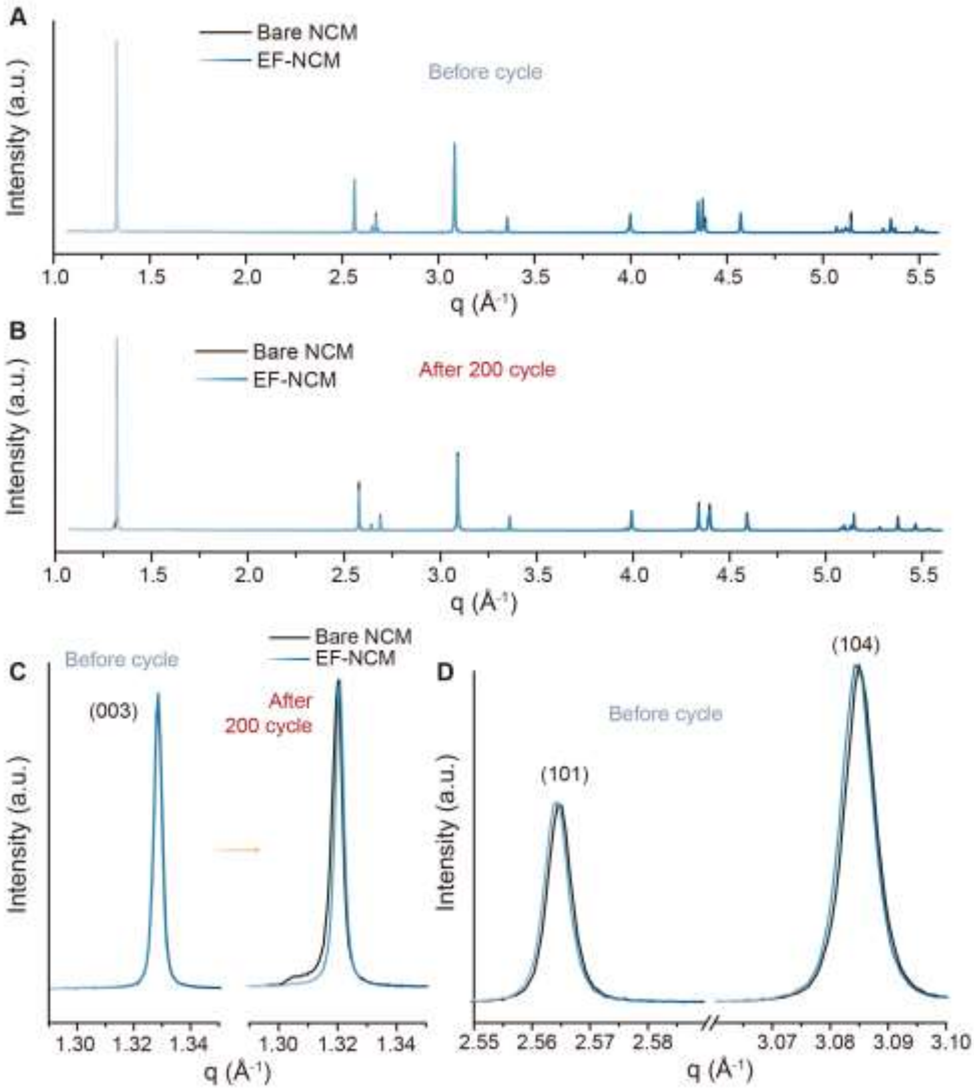

Figure S27. High-resolution powder diffraction (HRPD) analysis result of bare NCM and EF-NCM; (A) before the cycle, (B) after 200 cycles, (C) (003) peak of before and after 200 cycles, (D) (101)/(104) peak of before cycle.

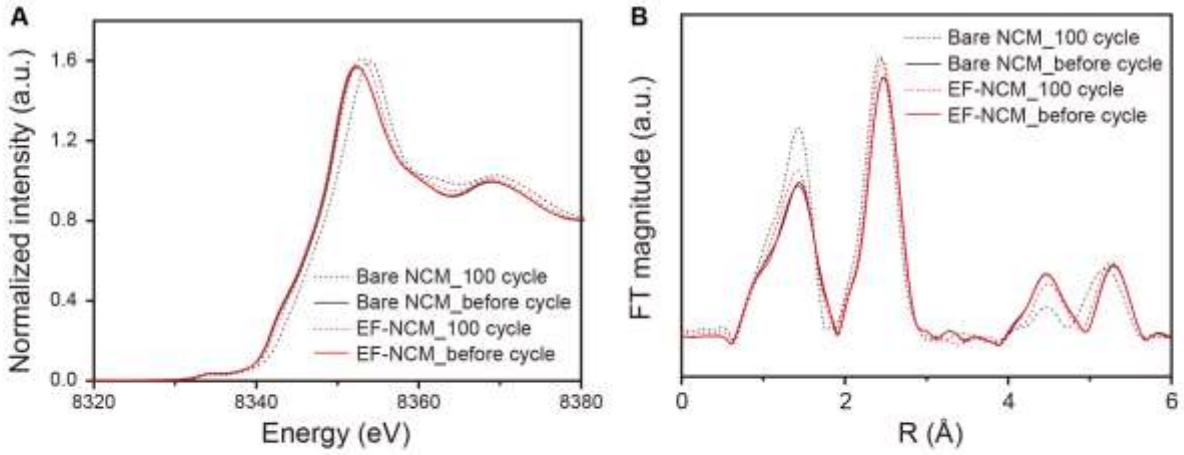

Figure S28. Nickel K-edge XAFS analysis result of bare NCM and EF-NCM after 100 cycles; (A) XANES, and (B) Extended X-ray absorption fine structure (EXAFS).

To observe changes in the valence state of nickel, XANES analysis was conducted (Figure S28A). The midpoint of the nickel edge between the edge rise and maximum was used to define the valence state.[13] The nickel K-edge of bare NCM after 100 cycles shifted to a higher energy state compared to that before cycling, indicating that the nickel was oxidized after cycling. In contrast, the electrode consisting of EF-NCM exhibited an almost identical valence state before and after 100 cycles, demonstrating the reversibility of EF-NCM compared to bare NCM. EXAFS analysis also revealed variations in the bonding structure before and after cycling (Figure S28B). The bare NCM showed a significant increase in magnitude after 100 cycles in the Ni-O bond compared to EF-NCM. This result is attributed to the soft Jahn-Teller effect of Ni^{3+} ,[14] meaning that bare NCM electrode have lower electrochemical reversibility.

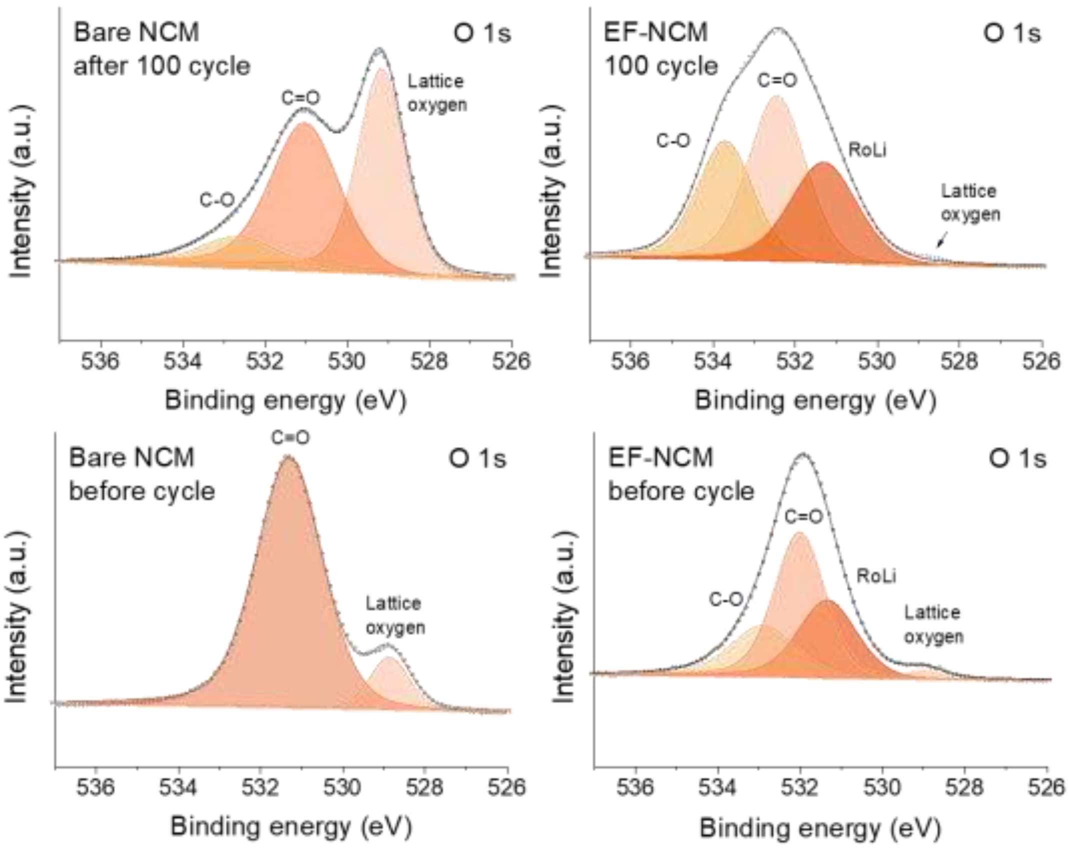

Figure S29. XPS O1s analysis result of bare NCM and EF-NCM.

For proving the effect of electrolyte decomposition, XPS analysis was conducted. Before cycling, bare NCM exhibited a distinct lattice oxygen signal (~ 529 eV) compared to EF-NCM, likely due to the surface decoration by the elastic framework. After 100 cycles, bare NCM still showed a clear lattice oxygen signal, indicating that the SEI layer thickness remained below 5 ~ 10 nm, consistent with the penetration depth of XPS analysis. EF-NCM also displayed a small lattice oxygen peak after 100 cycles.

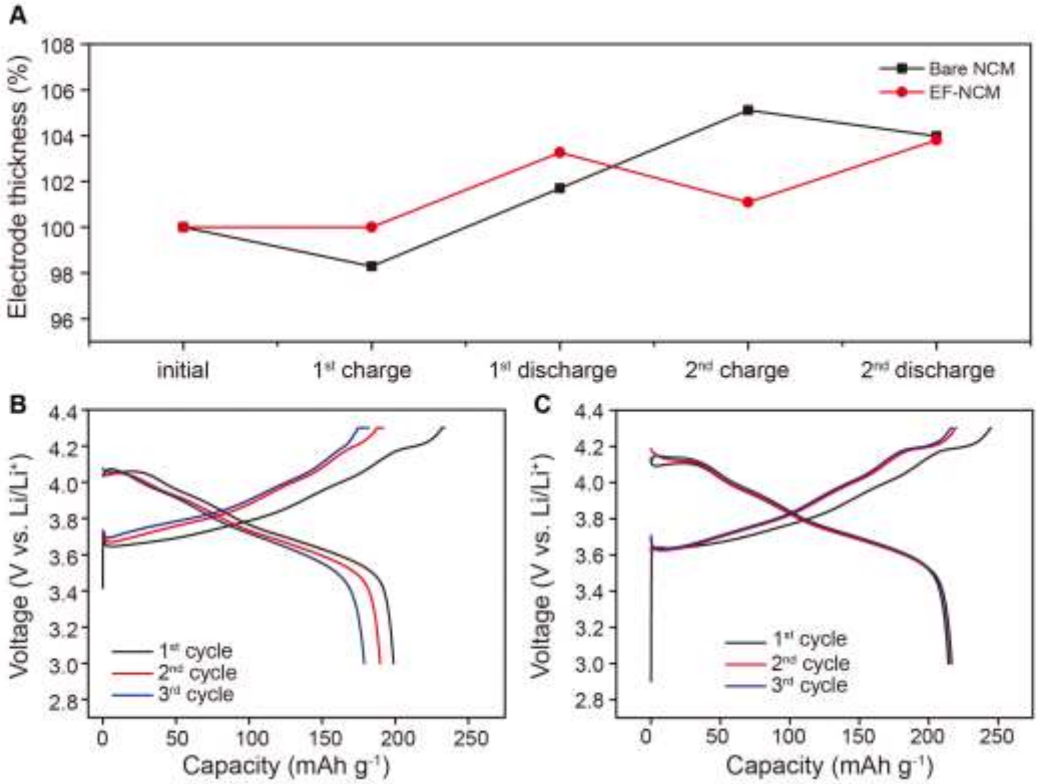

Figure S30. Thickness tracking result of high loading-low carbon electrode; (A) tracked electrode thickness through micrometer, (B) GCD curve of bare NCM, (C) GCD curve of EF-NCM.

The thickness of the electrode was tracked for cross-checking the thickness variation. Bare NCM and EF-NCM electrodes cycled with lithium metal anode were disassembled at each charge, discharge process in Ar atmosphere glove box, measuring the thickness through a micrometer. This result shows that the thickness variation of bare NCM was larger than EF-NCM (Figure S30A). As a result of large volume variation, GCD curve of bare NCM showed tremendous irreversibility compared to that of EF-NCM (Figure S30B, Figure S30C). Because of the properties of the thickness gauge, pressing the electrode in a vertical direction, the thickness value was different compared to the FE-SEM image.

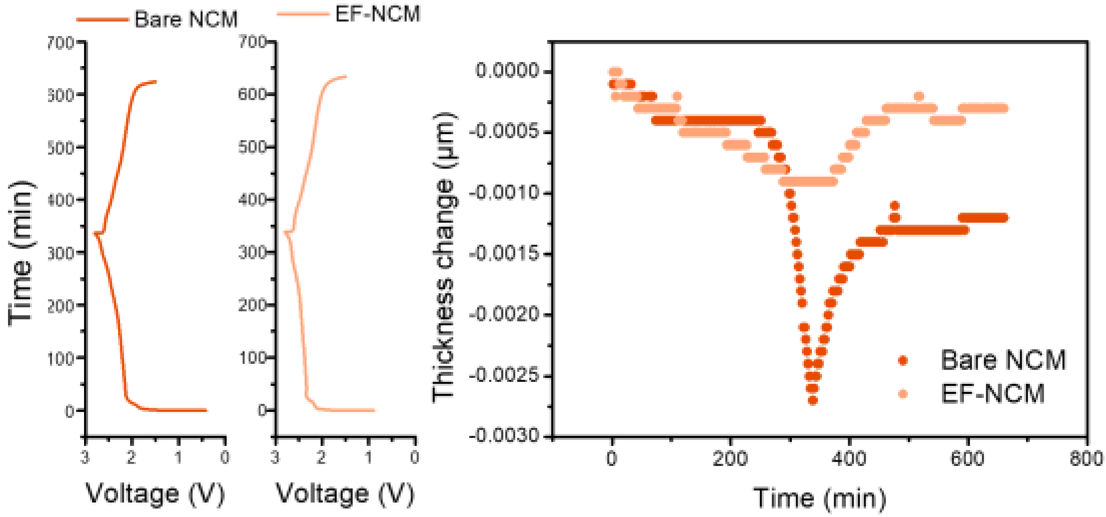

Figure S31. *In-situ* dilatometry result of bare NCM and EF-NCM

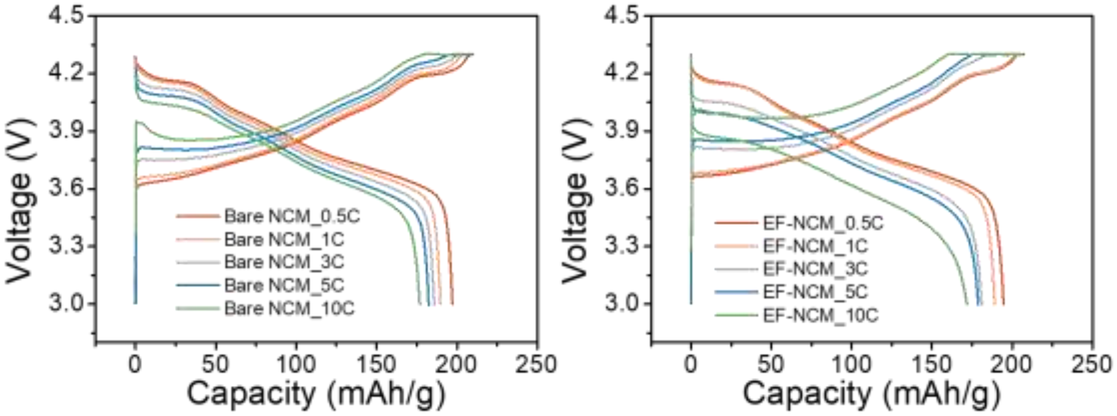

Capacity (mAh g^{-1})	0.5C	1C	3C	5C	10C
**Bare NCM**	197.20	189.71	185.75	182.44	176.78
**EF-NCM**	194.50	188.92	180.89	178.84	171.67

Figure S32. Charge discharge curve of bare NCM and EF-NCM at various C-rates.

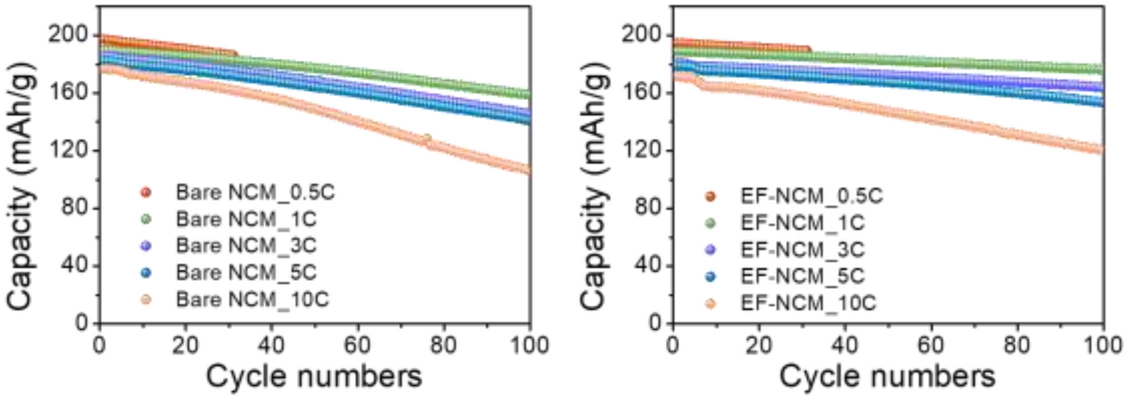

Capacity after 100 cycle (mAh g^{-1})	0.5C (30 cycle)	1C	3C	5C	10C
**Bare NCM**	186.54	158.78	145.48	140.85	105.94
**EF-NCM**	189.57	176.17	163.81	153.44	120.31

Figure S33. Cycle retention of bare NCM and EF-NCM at various rate magnification.

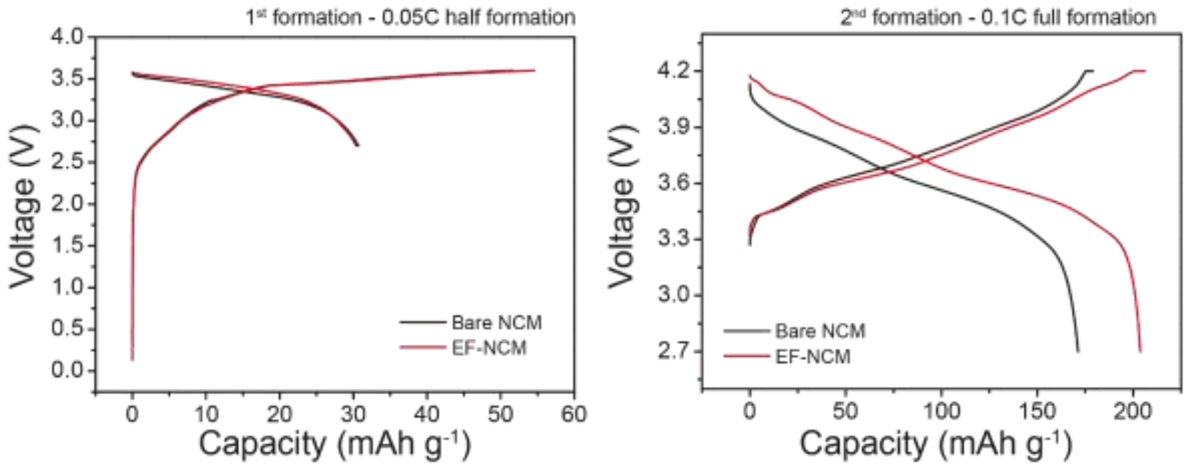

Figure S34. GCD curve of 1st half formation, and 2nd full formation. This electrode was fabricated with total 0.5 wt % conductive material (bare NCM - 0.5 wt % super C65, EF-NCM - 0.5 wt % elastic framework).

The fabricated pouch cell conducted 1st half formation at 0.05C and 2nd full formation at 0.1 C-rate for fabricating the stable SEI layer. At 0.05C 1st half formation cycle between voltage window 3.6 V to 2.7 V, bare NCM and EF-NCM showed similar capacity retention. However, at 0.1C 2nd full formation, bare NCM presented 170 mAh g^{-1}, almost 30 mAh g^{-1} lower than EF-NCM, it is anticipated that result of particle volume variation.

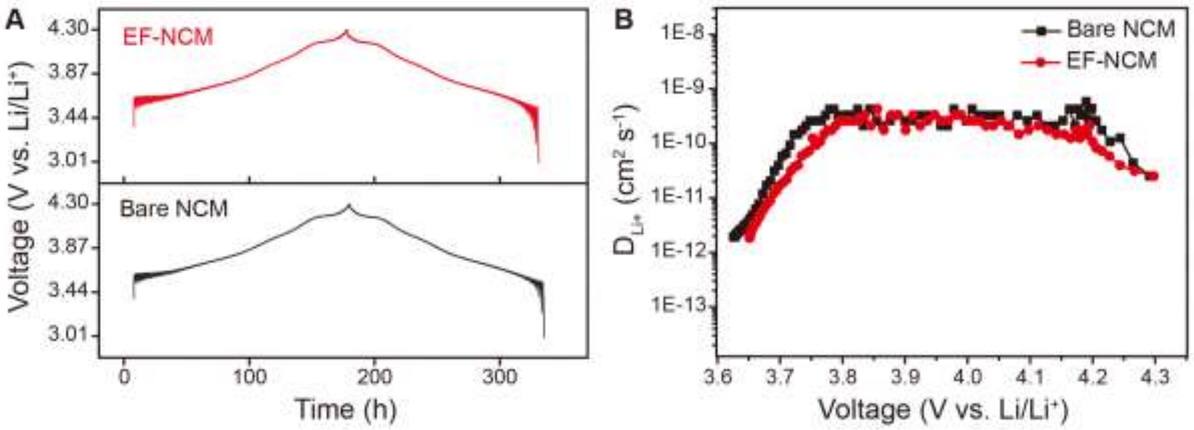

Figure S35. (A) GITT analysis result and (B) extracted diffusion coefficient parameter

The investigation into the variation of lithium-ion diffusion resulting from internal space was conducted by extracting the diffusion coefficient from the Galvanostatic Intermittent Titration Technique (GITT) results (Figure S35A). The diffusion coefficient was proposed based on the equation as follows;

$$D = \frac{4r^2}{\pi\tau}\left(\frac{\Delta E_s}{\Delta E_t}\right)^2$$

The presented equation was displayed through key parameters; r, representing size of active particles; ΔE_s, denoting change of the voltage at each step; ΔE_t, signifying voltage variation during applied time (τ); D, representing the diffusion coefficient. The current was applied to 600 s.[15] Within the voltage range of 4.1~4.3 V, associated with the H2-H3 phase transition, EF-NCM exhibited a comparable diffusion coefficient to bare NCM (Figure S35B).

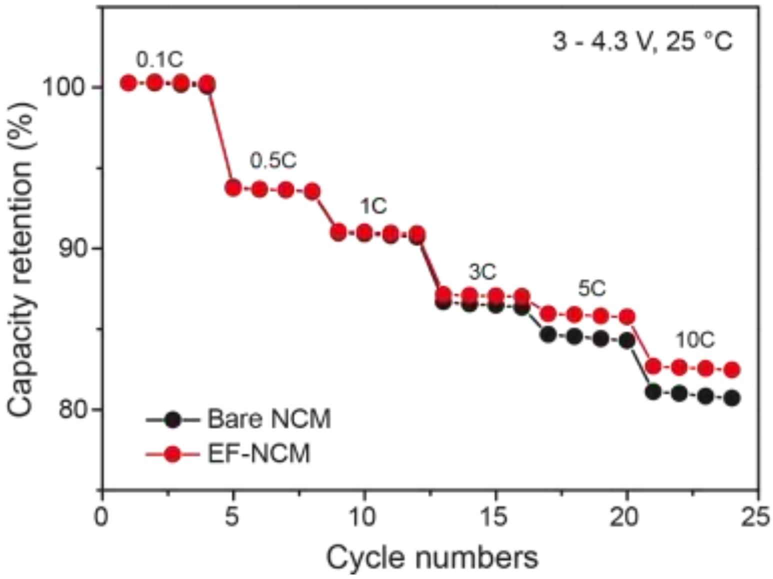

Figure S36. C-rate analysis result of bare NCM and EF-NCM

The rate properties exhibited similar behavior at 0.1, 0.5, 1, and 3 C-rates, with an increase observed at 5 and 10 C-rates. This finding implies that the volume contraction of primary particles and the increase in internal volume had a marginal influence on lithium-ion diffusion.

References

(1) Datsyuk, V.; Kalyva, M.; Papagelis, K.; Parthenios, J.; Tasis, D.; Siokou, A.; Kallitsis, I.; Galiotis, C. Chemical Oxidation of Multiwalled Carbon Nanotubes. *Carbon* **2008**, *46*, 833-840. DOI: https://doi.org/10.1016/j.carbon.2008.02.012.

(2) Li, J.; Li, Y.; Guo, Y.; Lv, J.; Yi, W.; Ma, P. A Facile Method to Enhance Electrochemical Performance of High-Nickel Cathode Material $Li(Ni_{0.8}Co_{0.1}Mn_{0.1})O_2$ *via* Ti Doping. *J. Mater. Sci.: Mater. Electron.* **2018**, *29*, 10702-10708. DOI: https://doi.org/10.1007/s10854-018-9093-1.

(3) Dresselhaus, M. S.; Dresselhaus, G.; Saito, R.; Jorio, A. Raman Spectroscopy of Carbon Nanotubes. *Phys. Rep.* **2005**, *409*, 47-99. DOI: https://doi.org/10.1016/j.physrep.2004.10.006.

(4) Jorio, A.; Saito, R. Raman Spectroscopy for Carbon Nanotube Applications. *J. Appl. Phys.* **2021**, *129*, 021102. DOI: 10.1063/5.0030809 (acccessed 3/29/2024).

(5) Cooper, C.; Young, R.; Halsall, M. Investigation into the Deformation of Carbon Nanotubes and Their Composites through the Use of Raman Spectroscopy. *Compos. - A: Appl. Sci.* **2001**, *32*, 401-411. DOI: https://doi.org/10.1016/S1359-835X(00)00107-X.

(6) Moon, J.; Jung, J. Y.; Hoang, T. D.; Rhee, D. Y.; Lee, H. B.; Park, M.-S.; Yu, J.-S. The Correlation between Particle Hardness and Cycle Performance of Layered Cathode Materials for Lithium-Ion Batteries. J. Power Sources **2021**, *486*, 229359. DOI: https://doi.org/10.1016/j.jpowsour.2020.229359

(7) Marinescu, I. D.; Doi, T.; Uhlmann, E. Handbook of Ceramics Grinding and Polishing; William Andrew, **2015**.

(8) Pajak, M.; Ponikiewski, T. Flexural Behavior of Self-Compacting Concrete Reinforced with Different Types of Steel Fibers. Construction and Building Materials **2013**, *47*, 397-408. DOI: https://doi.org/10.1016/j.conbuildmat.2013.05.072

(9) Hsieh, T.; Kinloch, A.; Taylor, A.; Kinloch, I. The Effect of Carbon Nanotubes on the Fracture Toughness and Fatigue Performance of a Thermosetting Epoxy Polymer. *J. Mater. Sci.* **2011**, *46*, 7525-7535 DOI: https://doi.org/10.1007/s10853-011-5724-0

(10) Tang, Xingling; El-Hami, A.; El-Hami, K.; Eid, M.; Si, C. Elastic Properties of Single-Walled Carbon Nanotube Thin Film by Nanoindentation Test. *Sci. Rep.* **2017**, *7*, 11438. DOI: https://doi.org/10.1038/s41598-017-11722-y.

(11) Li, S.; Xiong, R.; Han, Z.; He, R.; Li, S.; Zhou, H.; Yu, C.; Cheng, S.; Xie, J. Unveiling Low-Tortuous Effect on Electrochemical Performance Toward Ultrathick $LiFePO_4$ Electrode

with 100 mg cm^{-2} Area Loading. *J. Power Sources* **2021**, *515*, 230588. DOI: https://doi.org/10.1016/j.jpowsour.2021.230588.

(12) Choi, J.; Lee, C.; Park, S.; Embleton, T. J.; Ko, K.; Jo, M.; Saleem Saqib, K.; Yun, J.; Jo, M.; Son, Y. Analysis of Electrochemical Performance with Dispersion Degree of CNTs in Electrode According to Ultrasonication Process and Slurry Viscosity for Lithium-Ion Battery. *Nanomaterials* **2022**, *12*, 4271. DOI: https://doi.org/10.3390/nano12234271.

(13) Jo, S.; Han, J.; Seo, S.; Kwon, O. S.; Choi, S.; Zhang, J.; Hyun, H.; Oh, J.; Kim, J.; Chung, J. Solid□State Reaction Heterogeneity During Calcination of Lithium□Ion Battery Cathode. *Adv. Mater.* **2023**, *35*, 2207076. DOI: https://doi.org/10.1002/adma.202207076.

(14) Balasubramanian, M.; Sun, X.; Yang, X.; McBreen, J. *In-Situ* X□ray Absorption Studies of a High□Rate $LiNi_{0.85}Co_{0.15}O_2$ Cathode Material. *J. Electrochem. Soc.* **2000**, *147*, 2903. DOI: 10.1149/1.1393624.

(15) Liu, Q.; Su, X.; Lei, D.; Qin, Y.; Wen, J.; Guo, F.; Wu, Y. A.; Rong, Y.; Kou, R.; Xiao, X. Approaching the Capacity Limit of Lithium Cobalt Oxide in Lithium Ion Batteries *via* Lanthanum and Aluminium Doping. *Nat. Energy* **2018**, *3*, 936-943. DOI: https://doi.org/10.1038/s41560-018-0180-6
